# Single-copy locus proteomics of early- and late-firing DNA replication origins identifies a role of Ask1/DASH complex in replication timing control

**DOI:** 10.1016/j.celrep.2023.112045

**Published:** 2023-01-25

**Authors:** Matthias Weiβ, Anna Chanou, Tamas Schauer, Andrey Tvardovskiy, Stefan Meiser, Ann-Christine König, Tobias Schmidt, Elisabeth Kruse, Henning Ummethum, Manuel Trauner, Marcel Werner, Maxime Lalonde, Stefanie M. Hauck, Antonio Scialdone, Stephan Hamperl

**Affiliations:** 1 Institute of Epigenetics and Stem Cells, Helmholtz Zentrum München, Feodor-Lynen-Strasse 21, 81377 München, Germany; 2 Institute of Functional Epigenetics, Helmholtz Zentrum München, Ingolstädter Landstrasse 1, 85764 Neuherberg, Germany; 3 Institute of Computational Biology, Helmholtz Zentrum München, Ingolstädter Landstrasse 1, 85764 Neuherberg, Germany; 4 Metabolomics and Proteomics Core, Helmholtz Zentrum München, German Center for Environmental Health, Heidemannstrasse 1, 80939 München, Germany

**Keywords:** locus-specific chromatin isolation, site-specific recombination, affinity purification, DNA replication origin, replication timing, replication efficiency, origin chromatin structure, DASH complex, Ask1 protein, microtubule cytoskeleton

## Abstract

The chromatin environment at origins of replication is thought to influence DNA replication initiation in eukaryotic genomes. However, it remains unclear how and which chromatin features control the firing of early-efficient (EE) or late-inefficient (LI) origins. Here, we use site-specific recombination and single-locus chromatin isolation to purify EE and LI replication origins in *Saccharomyces cerevisiae*. Using mass spectrometry, we define the protein composition of native chromatin regions surrounding the EE and LI replication start sites. In addition to known origin interactors, we find the microtubule-binding Ask1/DASH complex as an origin-regulating factor. Strikingly, tethering of Ask1 to individual origin sites advances replication timing (RT) of the targeted chromosomal domain. Targeted degradation of Ask1 globally changes RT of a subset of origins, which can be reproduced by inhibiting microtubule dynamics. Thus, our findings mechanistically connect RT and chromosomal organization via Ask1/DASH with the microtubule cytoskeleton.

## Highlights

• Single-locus chromatin isolation shows distinct protein interactions at EE/LI origins
• Ask1/DASH complex can regulate replication timing of ∼450 chromosomal locations
• Ask1-activated regions are enriched for EE replication origins
• Ask1-repressed regions are preferentially located at LI and inter-origin locations

## Graphical abstract

**Figure:**
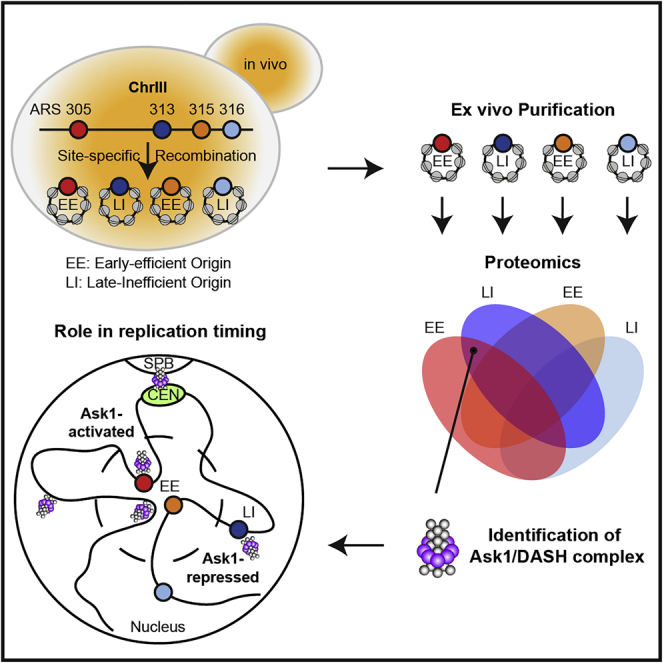

## Teaser

Weiβ et al. establish a system of native single-locus chromatin isolation in yeast to identify proteins associated with distinct early-efficient (EE) or late-inefficient (LI) replication origins. The results identify the microtubule-binding Ask1/DASH complex as an origin-regulating factor that connects chromosomal organization in the nucleus with replication timing control.

## Introduction

DNA replication is the fundamental process that duplicates genetic information once per cell cycle. The replication machinery initiates at specialized genomic regions called origins of replication.^1^^,^^2^ The *Saccharomyces cerevisiae* genome comprises ∼400 experimentally validated origins. Yeast origins are termed autonomously replicating sequences (ARSs) because of their ability to confer extrachromosomal plasmid replication.^3^ Unlike other eukaryotic origins, ARSs contain a specific 11 bp ARS consensus sequence (ACS), which was later increased to a 17 bp extended ACS sequence (eACS).^4^ Notably, other functional DNA elements named B1, B2, and B3 contribute to the activity of origins in a modular structure. The core B1 sequence contains WTW nucleotides from 17 to 19 bp 3′ to the ACS T-rich strand and serves as an ORC-binding site.^5^^,^^6^^,^^7^ In addition, eACS and B1 elements were recently shown to function as a binding site for an ORC-Cdc6 complex that facilitates the recruitment of MCM2-7 helicase.^8^^,^^9^ The B2 element contains a common ANWWAAAN sequence among 74% of annotated origins and is also suggested to support binding of MCM2-7.^10^^,^^11^ The B3 element is a transcription factor binding site for Abf1, which helps to position nucleosomes around a subset of replication origins including the well-characterized ARS1 origin.^12^^,^^13^ Interestingly, significant variability in the presence of these DNA sequences exists and not every origin utilizes the complete set of B elements, suggesting that their modular presence at origins is auxiliary and promotes origin function, but is not an essential feature of an active chromosomal replicator.

Similar to higher eukaryotes, origins in yeast differ drastically in their replication timing and efficiencies.^14^^,^^15^ Much progress has been made to elucidate underlying principles behind these differences. For example, the majority of ARSs show characteristic patterns of nucleosome occupancy, where ORC binding induces a nucleosome-free region flanked by well-positioned nucleosomes around the ARSs.^16^^,^^17^^,^^18^ Disruption of this ORC-directed nucleosome phasing interferes with efficient replication initiation,^19^ suggesting that interactions of ORC with adjacent nucleosomes is likely to contribute to origin activity. In addition, local histone modifications have been suggested to direct replication timing (RT), as enrichment of histone H3- and H4-acetylation was detected after pull-down of a minichromosome containing the early-efficient (EE) origin ARS1.^20^ In agreement, deletion of Rpd3 histone deacetylase advances the RT of a subset of 10 early- and late-firing origins,^21^^,^^22^ whereas artificial recruitment of histone acetyltransferase Gcn5 advances the RT of a late-firing origin (ARS1412).^22^

Chromosomal context is another important aspect of origin regulation. For example, subtelomeric origins adopt a silent chromatin state and generally fire late in S-phase, whereas centromeric regions are early replicating.^23^ Subtelomeres are highly enriched for Rif1, which acts as a global negative regulator of RT.^24^^,^^25^ Thus, cloning late-replicating subtelomeric ARS into an extrachromosomal plasmid confers early replication status to the plasmid,^26^ suggesting that Rif1-mediated repression at chromosomal ends inhibits the default state of early RT of such origins. However, a specific “late” DNA sequence element present on an internal late-inefficient (LI) origin ARS1412 can retain late RT even when inserted on a plasmid,^27^ supporting the view that alternative, non-mutually exclusive DNA- and chromatin-based mechanisms are in place at individual origins. Another important aspect is the 3D nuclear organization of origins.^28^^,^^29^ Seminal DNA-FISH experiments showed that LI origins associate with the nuclear periphery in G1-phase, whereas EE origins cluster in the nuclear center.^30^ More recently, it was shown that this localization is dependent on the forkhead transcription factors Fkh1/Fkh2, which bind to a subset of Fkh-activated EE origins and promote nuclear relocalization and clustering of replication factories.^31^ Together, these studies indicate that several parameters, including nucleosome occupancy, histone modifications, non-histone chromatin-associated proteins, and chromosomal context, as well as spatial organization of origins, contribute or likely work together to modulate origin behavior. However, the precise chromatin interactome and structural features that allow efficient and timely replication initiation at individual origins remain largely elusive.

Analyzing single-locus proteomes of defined chromosomal domains that encompass EE or LI replication origins would provide great insights into the chromatin interactions at such distinct classes of replication origins. Despite ongoing efforts for many years, single-locus chromatin proteomics still poses a major biochemical challenge owing to the small size of target loci compared with the large genomic background.^32^ We have previously established an approach for proteome investigation of repetitive loci based on site-specific recombination to purify selected chromosomal domains from yeast.^33^^,^^34^ Here, we have optimized this approach to investigate the proteome of four distinct single-copy replication origins with major differences in their RT. Besides expected interactors of general chromatin and known replication factors, we identify the Ask1/DASH complex as a factor regulating RT. Tethering Ask1 in vicinity to individual origins advances replication of the respective origin, demonstrating that Ask1 recruitment is sufficient to drive RT changes. Genome-wide RT analysis after degradation of Ask1 induces locally altered RT of a subset of origins as well as inter-origin regions. Ask1/DASH’s canonical function is to connect microtubules with the kinetochore. Perturbing microtubule dynamics by nocodazole treatment shows similar effects on RT as Ask1 degradation, suggesting that the effect of Ask1 on RT is dependent on its canonical function to bind microtubules. These results suggest a specific class of Ask1-dependent replication origins that connect specific chromosomal regions with the nuclear cytoskeleton. Thus, our unbiased approach can characterize the local origin chromatin structure of specific genomic loci and can identify important functional interactions that affect the capacity of these regions to initiate replication.

## Results

### DNA sequence elements of yeast origins do not correlate with their RT and efficiency

**Figure 1:**
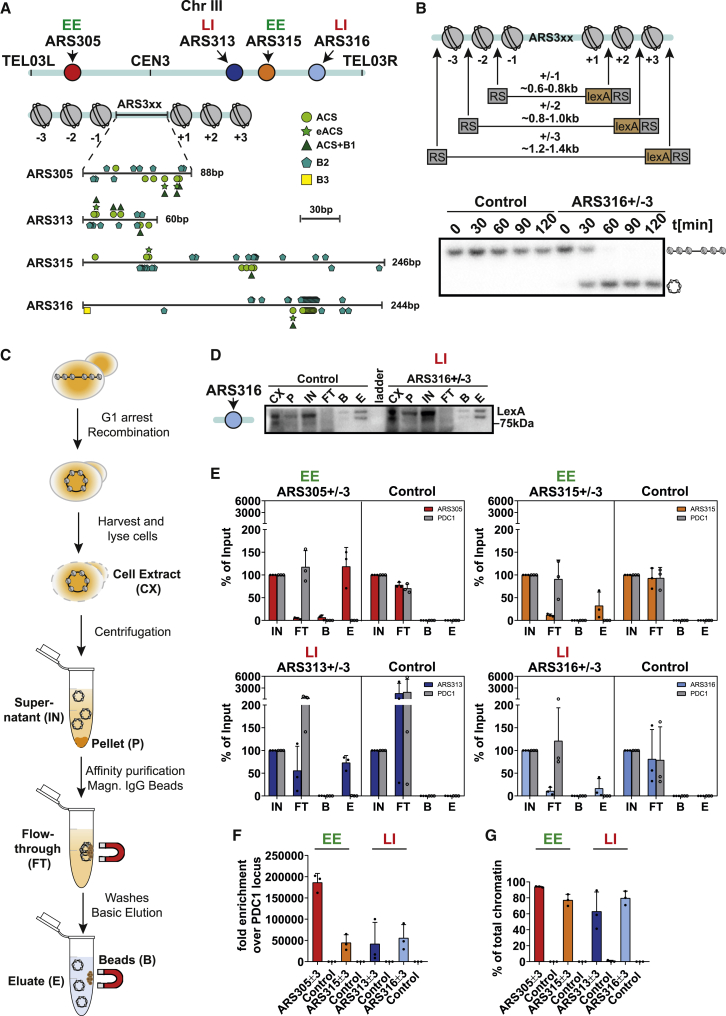
Distinct EE and LI replication origins are purified with high yield and purity (A) Chromosomal features of EE and LI origins on chromosome III. Cartoon shows annotated ACS, eACS, B1, B2, and B3 elements at origins. (B) For each origin, strains were created with integrated recombination sites after the first (+/−1), second (+/−2), or third (+/−3) pairs of nucleosomes around origins. Lower panel: yeast strains Y0066 (Control) and Y0069 (ARS316+/−3) were arrested in G1-phase with addition of 2% galactose to induce recombination. Genomic DNA samples at the indicated time points were linearized with BstBI and analyzed by Southern blot against ARS316 locus. (C) Experimental outline for the single-locus chromatin purification system. (D) LexA affinity purification of yeast strains Y0069 (ARS316+/−3) and Y0066 (Control). Western blot against LexA of protein samples shown in (C): 0.1% for cell extract (CX), pellet (P), input (IN), and flowthrough (FT), and 1% for beads (B) and elution (E) (n = 1 biological replicate). (E) LexA affinity purifications of yeast strains Y0065 (ARS305+/−3), Y0091 (ARS315+/−3), Y0094 (ARS313+/−3), Y0069 (ARS316+/−3), and control strain (Y0066). DNA samples (0.1% for CX, P, IN, and FT, and 2.5%) for (B and E) were analyzed by qPCR to monitor enrichment of targeted origins and unrelated genomic region (PDC1). Bar plots with error bars indicating standard deviation from n = 3 biological replicates. (F) Fold enrichment of origin DNA compared with PDC1 in eluate samples. Bar plots with error bars indicating standard deviation from n = 3 biological replicates. (G) Proportion of total origin-derived DNA (∼1 kb) as a percentage of total chromatin by factoring in the size of total yeast genome (∼12,000 kb). Bar plots with error bars indicating standard deviation from n = 3 biological replicates. See also Figures S1–S5.

We focused our efforts on four selected origins on yeast chromosome III, which show an intrachromosomal position with >40 kb genomic distance to telomeric and centromere regions and major differences in their RT and efficiency properties. ARS305 and ARS315 are early replicating and efficient origins (EE), whereas ARS313 and ARS316 represent late-replicating and inefficient origins (LI) (Figure 1A). Origins also differ in the size of nucleosome-free regions and the distribution and number of ACS, eACS, B1, B2, and B3 elements (Figure 1A). As most of these DNA elements interact with the core licensing machinery, a primary function of these sequences could be to efficiently guide ORC and MCM complexes during origin licensing in G1-phase. Therefore, we first set out to systematically characterize the abundance of ACS, eACS, B1, B2, and B3 consensus motifs in all 352 annotated origins (Table S1). We scanned each ARS and counted the occurrence of these five consensus motifs, allowing for up to two mismatches for each motif instance (Figure S1A). In addition, we included the flanking 200 bp upstream and downstream of ARSs for motif searches of B3 elements, as Abf1 binding outside of nucleosome-free regions was described previously at specific origins such as ARS318.^5^

We asked whether the presence of these motifs might affect origin properties. To this end, we correlated publicly available RT data for all 352 origins^35^ and 215 origins where replication efficiency data were available^14^ with the number of single DNA motifs at each origin. We found no statistically significant correlations with any of the tested DNA motifs (Figures S1B and S1C). Based on this analysis, we conclude that timing and efficiency properties of origins are unlikely to be dictated by DNA elements, strengthening our hypothesis that the local chromatin structure or other factors may be more decisive than DNA sequence properties.

### Distinct EE and LI replication origins are purified with high yield and purity

To investigate interactomes of specific origins, we next constructed libraries of yeast strains where each selected ARS was tagged with site-specific recombination sites (RSs) and LEXA binding sites. RS/LEXA cassettes were inserted at increasing distances from the ARS, such that excised circular chromatin domains include the first one, two, or three pairs of nucleosomes centered around ARSs (Figure 1B).

It is critical to define ARS chromatin in G1-phase, as this represents the *bona fide* substrate for replication initiation in the subsequent S-phase. Therefore, we arrested cells competent for origin recombination in G1-phase and simultaneously induced R-recombinase expression. Successful G1-arrest was verified by fluorescence-activated cell sorting (FACS) (Figures S2A and S2B). Recombination kinetics were monitored in a time course experiment by Southern blotting of extracted genomic DNA. In all strains analyzed, we observed near complete recombination of target loci within ∼60–90 min after recombinase induction (Figures 1B, S2C, and S2D). Kinetics of circularization were independent of the size of excised domains, suggesting that topological constraints were not rate-limiting even in the smallest domain encompassing +/−1 nucleosomes (Figure S2D).

Initial attempts to retrieve target chromatin using original constructs and protocols suffered from low yields and high contamination from genomic DNA and non-specific proteins. To improve yield and purity of chromatin domains, we replaced the original weak promoter driving LexA-TAP expression with a stronger, constitutive promoter. This modification increased LexA-TAP levels by ∼5- to 6-fold (Figures S3A and S3B) and improved retention and purity of origin chromatin (Figures S3C and S3D).

Importantly, we verified that our genetic manipulations did not interfere with origin function by comparing the RT of the origins in recombination strains with an isogenic control strain, using DNA copy-number analysis by quantitative PCR (qPCR). Cells were arrested in G1-phase and synchronously released into S-phase. Copy-number analysis of early-replicating ARS305 versus late-replicating ARS316 or a late-replicating region on chromosome IV (Chr4^36^) showed that both ARS305 and ARS316 loci replicated with highly similar kinetics in wild-type and modified strains (Figures S4A and S4B). We also verified that recombination, which involves transient DNA breaks, did not elicit measurable DNA damage responses as recombination of ARS305 and re-ligation of genomic ends showed no detectable global increase of ɣH2A phosphorylation (Figures S5A and S5B).

We focused our biochemical purifications on ARS3xx+/−3 strains to isolate origins in the context of larger chromatin domains (Figure 1B). These ∼1.2–1.4 kb chromatin circles were purified by LexA-TAP mediated one-step affinity purification (Figure 1C). As negative control, we performed purifications from an isogenic strain that expresses LexA-TAP but lacks integrated RS and LEXA-binding sites. Western blot analysis of LexA-TAP in purification samples showed near-complete depletion of LexA-TAP in the flowthrough and recovery in the final elution as expected (Figures 1D and S5C). We quantified enrichment of origin DNA in these fractions by qPCR. Between ∼20% and 100% of target origins were recovered in recombination strains, but no enrichment in the control strain was observed. Another unrelated single-copy gene locus (PDC1) was similarly lost in both origin and control purifications, indicating specificity of our purification (Figure 1E). Indeed, elutions showed 30,000- to 150,000-fold origin enrichment over PDC1 (Figure 1F), corresponding to 15- to 20-fold excess over any other genomic DNA in the purified material. Therefore, we estimate that ∼60%–90% of all DNA molecules in these samples were derived from target loci (Figure 1G).

### LexA affinity purification identifies distinct proteomes at EE and LI origins

Having established specificity and sufficient yield of our approach, we next determined protein composition of individual origins using quantitative mass spectrometry (MS). To this end, we compared proteomes derived from each EE and LI origin with negative control purifications. For each origin, we achieved similar proteome coverages, with ∼1,500–3,000 proteins identified with more than 2 unique peptides from 3 biological replicates (Table S2). To reduce background contaminants, we only considered protein factors at least 1.4-fold enriched over control purifications. This arbitrary threshold resulted in 94, 29, 635, and 25 putative ARS-interacting proteins with ARS305, ARS315, ARS313, and ARS316, respectively, which we considered for subsequent analysis.

Nucleosomes, ORC, and MCM2-7 double-hexamer complexes are expected as the most abundant factors at licensed origins.^37^ To compare MS results with endogenous chromatin binding of these complexes, we profiled the chromatin structures of the four origins using chromatin immunoprecipitation (ChIP)-Exo datasets of canonical histones (HTA2, HTB2, HHF2, and HHT1), linker histone H1 (HHO1), ORC subunits (ORC1 and ORC4), and MCM2-7 (MCM5), and selected histone modifications associated with active transcription (H3K4me3) and decondensed accessible chromatin (H3K9ac and H4K12ac)^38^ (Figures S6A–S6D). As expected, nucleosome depletion at ARSs was clearly visible in histone profiles and overlapped strongly with ORC and MCM ChIP-Exo peaks. We noticed that the EE origins ARS305 and ARS315 showed a more prominent decrease of nucleosome occupancy around ARSs (Figures S6A and S6C), whereas canonical histones at the two LI origins ARS313 and ARS316 were more evenly distributed, indicative of more regular nucleosome occupancy (Figures S6B and S6D). Interestingly, ARS313 was largely devoid of H3K4me3 and characterized by lower levels of H3K9ac and H4K12ac compared with the other three origins, suggesting that this origin likely contains transcriptionally less active and more condensed chromatin (Figure S6D).

**Figure 2:**
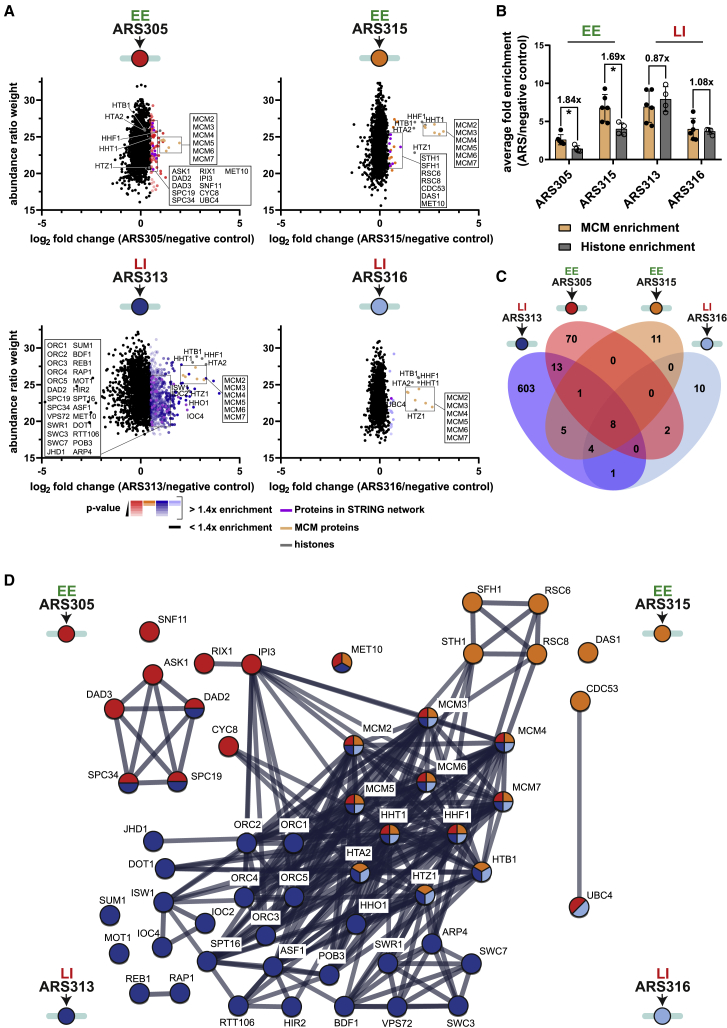
LexA affinity purification identifies distinct proteomes at EE and LI origins (A) Scatterplots of abundance ratio weights versus the average log2-fold enrichment of proteins at each replication origin purification (n = 3 biological replicates). MCM2-7 complex is shown in orange, histones in gray, and selected protein factors in purple. All proteins statistically enriched at least 1.4-fold over the control purification are colored according to origin and decreasing p value. (B) Bar plots of average enrichment of canonical histones (H2A, H2B, H3, and H4) and MCM2-7 subunits (MCM2, MCM3, MCM4, MCM5, MCM6, and MCM7) over control purifications (n = 3 biological replicates, ^∗^p < 0.05, unpaired t test). (C) Venn diagram of overlapping proteins detected at the four origins. (D) STRING network analysis of selected proteins at least 1.4-fold enriched over negative control. Individual proteins are shown as nodes, edges indicate interactions retrieved from STRING database (interaction score > 0.9). Significantly enriched proteins are colored according to the four origins. See also Figures S6–S8.

In agreement with these ChIP-Exo profiles, histones and all MCM2-7 subunits were highly enriched in origin purifications (Figures 2A and 2B). Notably, we observed highest abundance of histones at ARS313, the only origin where we could additionally detect the linker histone Hho1 (Figures 2A and 2B). Interestingly, EE origins ARS305 and ARS315 showed 1.84- or 1.69-fold higher relative enrichment of MCM molecules over histones, whereas the two LI origins showed no significant difference in MCM and histone levels (Figure 2B). Although our proteomic data do not allow differentiating between single loaded MCM2-7 complexes versus MCM2-7 double hexamers, this difference in MCM stoichiometry is consistent with previous reports that early origins can load multiple MCM double hexamers.^39^^,^^40^

To confirm these results by an independent method, we performed ChIP against an HA-tagged allele of MCM2 (HA-MCM2) and canonical histone H3. Importantly, this analysis was done on intact chromosomes without recombination or prior biochemical purification of chromatin. Consistent with MS results (Figure 2B), the two EE origins showed higher levels of HA-MCM2 compared with the LI origins (Figure S7A), whereas H3 was particularly enriched at the LI origin ARS313 (Figure S7B). We conclude that the investigated EE origins show higher MCM:histone ratios compared with the LI origins and this chromatin feature is preserved during recombination and purification steps.

We next intersected the four proteomes to identify common hits that could potentially promote EE versus LI replication origin status. Surprisingly, we observed limited overlap between factors enriched at both EE origins ARS305 and ARS315 (9 out of 114 proteins) as well as factors shared between the two LI origins ARS313 and ARS316 (13 out of 647 proteins) (Figure 2C). We note that five of six ORC subunits were detected at ARS313, but not in other origin purifications (Figure 2A). This could be caused by the stringent washing steps at 200 mM KCl salt concentration that may cause loss of more transient interactors as described for yeast ORC,^41^ or the fact that we did not complement the binding buffer with ATP shown to stabilize ORC binding.^42^

To increase our ability to purify transient, low-affinity interactions, we repeated all origin purifications under more physiological 150 mM KCl salt concentration. Importantly, we obtained similar enrichment levels of origin DNA as the purifications with 200 mM KCl (Figures S8A, S6B, 1F, and 1G), suggesting that these “low-salt” conditions did not impact the specificity of our purifications on the DNA level. We therefore proceeded with MS. The results show that we could not only reproduce strong enrichment of histones and MCM2-7 complexes (Figure S8C), but similarly obtained higher relative enrichment of MCM molecules over histones at EE origins ARS305 (1.45-fold) and ARS315 (1.21-fold) over LI origins ARS313 (1.07-fold) and ARS316 (0.90-fold) (Figure S8D). Interestingly, lowering the salt concentration to 150 mM KCl did not drastically improve the number of overlapping proteins at EE origins (27 out of 101 proteins) nor LI origins (13 out of 920 proteins) (Figure S8E). Furthermore, consistent with the results under high-salt conditions, three of six ORC subunits were detected at ARS313, but not for the other three origins. Together, these results suggest that the four isolated proteomes are highly distinct from each other, with each chromatin domain showing a distinct set of protein interactions.

We focused on proteins enriched under the more stringent high-salt condition to test the validity of the identified interactions. We prioritized protein complexes identified with multiple subunits on the same origin. Specific factors found at ARS305 (EE) included two subunits of the RIX complex (Rix1 and Ipi3) previously described to facilitate pre-RC formation.^43^ Interestingly, ARS305 also interacted with five subunits of the DASH complex (Ask1, Dad2, Dad3, Spc19, and Spc34), a large essential complex that connects the outer kinetochore to the mitotic spindle.^44^^,^^45^^,^^46^ Besides canonical histones and MCMs, the other EE origin ARS315 showed few other specific interactions including the SCF complex subunit Cdc53 together with the F box protein Das1. Strikingly, we also found four subunits of the RSC chromatin remodeling complex (Rsc6, Rsc8, Sth1, and Sfh1) at ARS315. A common interactor between the EE origins ARS305 and ARS315 was the sulfite reductase subunit Met10, which was identified in high-throughput screens as a negative genetic interactor with several RIX, MCM, and ORC complex subunits^47^ (Figures 2B–2D).

Similar to ARS315, only few specific interactions were identified at the LI origin ARS316 including the E2 enzyme Ubc4. Canonical histones and MCM proteins appeared to be the major chromatin components of this LI origin. This was in strong contrast to the second LI origin ARS313, where we identified ∼10-fold more specific proteins than for all other origins. This was a reproducible result under both low-salt and high-salt conditions (Figures 2A, 2C, S8C, and S8E; Table S2), indicating a larger complexity of interacting proteins at this origin (see discussion). Interestingly, ARS313 interacted with all subunits of the Isw1b complex (Isw1, Ioc2, and Ioc4) required to establish regular arrays of phased nucleosomes.^48^ We also identified nucleosome assembly factors and histone chaperones such as Asf1, Rtt106, Hir2, as well as both subunits of FACT complex (Spt16 and Pob3), consistent with the notion that this LI origin may exist in a more condensed, regular nucleosomal structure. In addition, six subunits of the SWR1 chromatin remodeling complex were found (Arp4, Bdf1, Vps72, Swr1, Swc3, and Swc7) as well as transcription factors such as Rap1, Reb1, Sum1, and Mot1. Intriguingly, ARS313 also interacted with three subunits of the DASH complex (Dad2, Spc19, and Spc34). As Ask1/DASH was already identified at ARS305 and thus appeared with multiple subunits in two of four origin purifications, we further investigated a potential role of this complex in origin regulation.

### Targeted recruitment of the DASH complex subunit Ask1 advances RT of specific chromosomal domains

The Ask1/DASH complex is a microtubule- and kinetochore-associated complex required for proper chromosome segregation and bipolar attachment of sister chromatids on the mitotic spindle^44^^,^^45^^,^^46^ and has not been reported previously in the context of replication origin chromatin. Interestingly, temperature-sensitive *ask1-2* and *ask1-3* mutants showed sensitivity to replication stress by hydroxyurea (HU) treatment (Figure S7C), implying a possible role in DNA replication.

**Figure 3:**
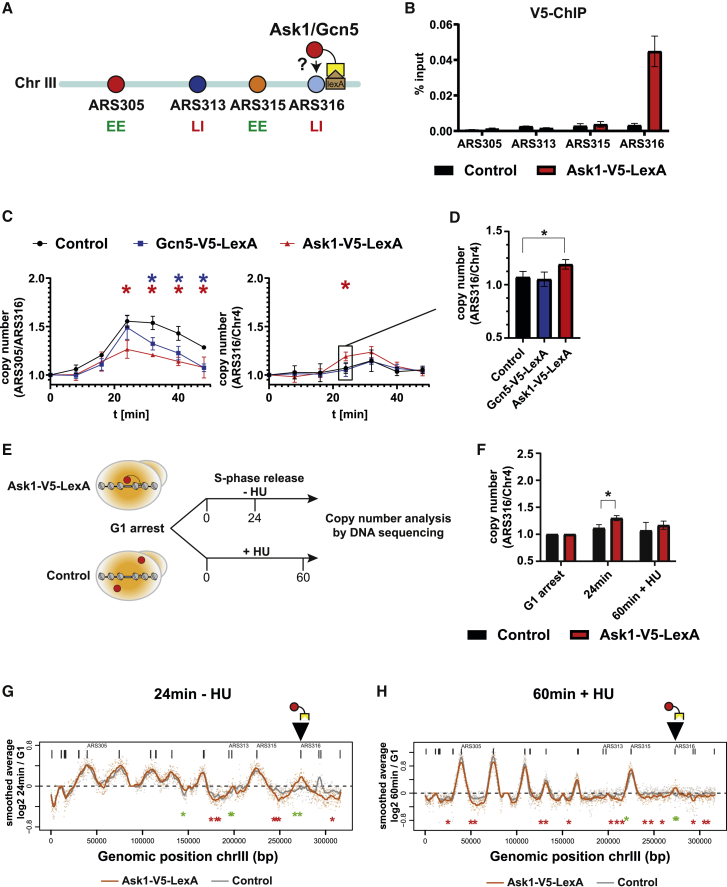
Recruitment of Ask1/DASH advances RT of LI origin ARS316 (A) Experimental outline for tethering Ask1/Gcn5 at LI origin ARS316. Yeast strains Y0071 and Y0079 express Ask1 or Gcn5 as LexA-V5 fusion proteins, respectively. (B) ChIP-qPCR using V5-antibodies to immunoprecipitate Ask1-V5-LexA at the indicated genomic regions in Ask1-V5-LexA (Y0071) and untagged control strain (Y0001). Bar plots with error bars indicating standard deviation from n = 3 biological replicates. (C) RT analysis of yeast strains Y0071(Ask1-V5-LexA), Y0079 (Gcn5-V5-LexA), and Y0001 (control strain). Samples for copy-number analysis were taken at the indicated time points. Plots show copy-number ratios of ARS305 and ARS316 to late-replicating region (Chr 4) with error bars indicating standard deviation from n = 3 biological replicates,^∗^p < 0.05, unpaired t test. (D) Bar graph of 24 min time point of ARS316/Chr4 ratio from (C). (E) RT analysis by copy-number DNA sequencing in yeast strains Y0071(Ask1-V5-LexA) and Y0019 (control). Cells were released into S-phase for 24 min in the absence or 60 min in the presence of 200 mM HU. (F) Genomic DNA samples from G1 arrest, 24 min release and 60 min + HU release were analyzed by qPCR. Plots show average copy-number ratios of ARS316 to late-replicating region (Chr 4). Bar plots with error bars indicating standard deviation from n = 3 biological replicates,^∗^p < 0.05, unpaired t test. (G) Replication profiles of chromosome III at 24 min after release into S-phase in the same strains as in (E). (H) Replication profiles of chromosome III at 60 min release into S-phase and 200 mM HU treatment in the same strains as in (G). See also Figures S7 and S9.

To determine whether Ask1 recruitment to LI origins can affect origin firing directly, we expressed Ask1 as a C-terminal LexA-V5 fusion protein in the ARS316 ± 1 strain, where LEXA binding sites were inserted ∼200 bp next to ARS316 (Figure 3A). V5 ChIP-qPCR revealed enrichment of Ask1-V5-LexA at ARS316 in contrast to an untagged control strain (Figure 3B), indicating successful recruitment of Ask1 to ARS316. Next, cells were arrested in G1-phase and synchronously released to S-phase to monitor RT of ARS316 by qPCR. In this analysis, the copy number of the EE origin ARS305 increased from 0 to 24 min after release, reflecting the earlier replication status compared with the LI origin ARS316. After 24 min, replication also initiates from LI regions and copy-number ratios decrease until S-phase is completed (Figure 3C, control). As positive control, we included a strain expressing Gcn5 as LexA-V5 fusion protein, as tethering of this protein was shown previously to advance replication of a late origin.^22^ Gcn5 recruitment caused a modest decrease of the copy-number ratio between ARS305 and ARS316 compared with the control strain, suggesting that ARS316 replication was slightly more efficient upon Gcn5 recruitment (Figure 3C, left panel). However, Ask1-LexA-V5 showed a much stronger decrease in the copy-number ratio of ARS305 to ARS316, suggesting that the RT of ARS316 was substantially advanced (Figure 3C, left panel). We obtained similar results when comparing copy-number ratios of ARS316 with another late-replicating region (Chr4), where Ask1 recruitment—but not recruitment of Gcn5—significantly advanced ARS316 replication (Figures 3C, right panel, and 3D).

To confirm these results and extend the analysis to the entire chromosome, we monitored DNA replication in the same strains by DNA copy-number sequencing.^49^^,^^50^ In brief, cells arrested in G1-phase were synchronously released into S-phase for 24 min to allow both initiation and elongation of DNA replication. In addition, we released cells in the presence of 200 mM HU for 60 min to allow initiation of early origins but restrict elongation to a ∼5 kb region around EE origins.^51^ Cells were collected in G1, 24 min, and 60 min + HU conditions. Genomic DNA was analyzed by qPCR and DNA sequencing (Figure 3E). qPCR analysis reproduced a significant increase of DNA copy number at Ask1-V5-LexA-targeted ARS316 24 min after release compared with control cells, whereas 60 min + HU samples showed a similar but less pronounced tendency (Figure 3F). Importantly, recruitment of LexA-TAP as a control LexA-fusion protein gave identical RT profiles compared with control cells (Figure S9A), strongly suggesting that this effect on RT is specific to Ask1 recruitment.

The genome-wide replication profiles showed expected relative copy-number changes around active origins in both 24 min release and 60 min + HU conditions (Figures 3G and 3H). In Ask1-V5-LexA cells, we observed a marked increase in ARS316 replication relative to control cells (Figures 3G and 3H, black arrow), consistent with qPCR results (Figures 3D and 3F). Interestingly, an additional two regions on the right arm of chromosome III significantly advanced RT (Figure 3G, green asterisk), whereas three intervening late-replicating regions showed decreasing copy-number ratios (Figure 3G, red asterisks). Under 60 min + HU conditions, Ask1 recruitment to ARS316 showed a small but significant increase in DNA copy number at the target origin (Figure 3H, black arrow), suggesting that a population of cells shifted ARS316 replication into early S-phase. Like the 24 min samples, neighboring EE origins including ARS315 advanced replication (Figure 3H, green asterisk), whereas multiple late-replicating regions scattered along chromosome III were further delayed (Figure 3H, red asterisk). Together, these data show that Ask1 tethering to the LI origin ARS316 is sufficient to advance replication of this origin as well as neighboring EE regions at the cost of less-efficient replication of intervening LI regions. These data are consistent with a model that Ask1/DASH recruitment at specific chromosomal sites can rearrange or cluster larger chromosomal regions in a way to enlarge RT differences between EE and LI regions.

**Figure 4:**
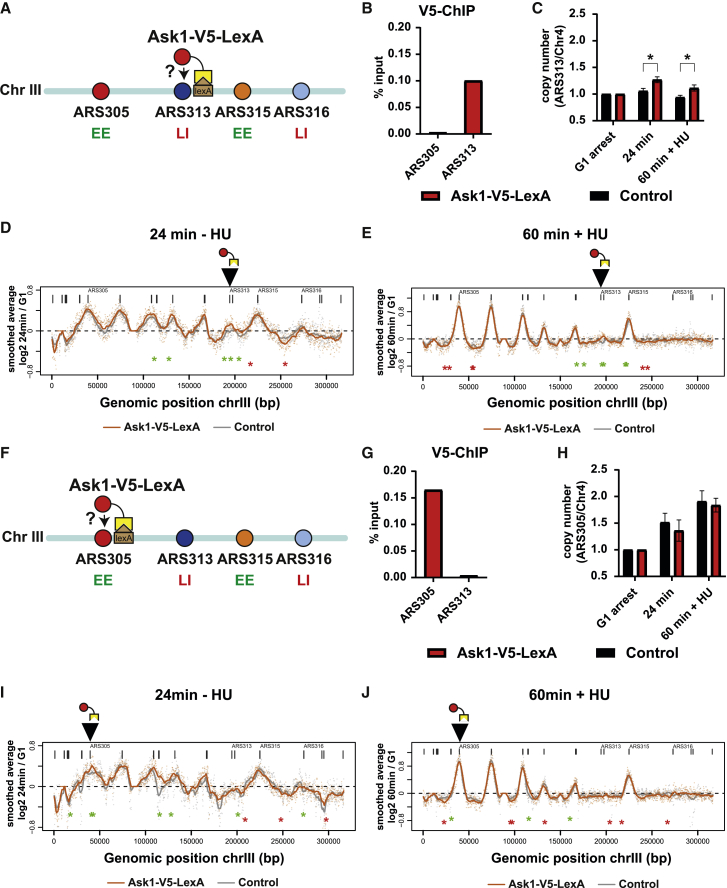
Recruitment of Ask1/DASH advances local replication of neighboring EE regions and delays replication of intervening LI regions (A) Experimental outline for tethering Ask1 at ARS313. Yeast strain Y0139 expresses Ask1 as LexA-V5 fusion protein for artificial recruitment of Ask1 to ARS313. (B) ChIP-qPCR analysis using V5-antibodies to immunoprecipitate Ask1-V5-LexA at the indicated genomic regions (n = 1 biological replicate). (C) RT analysis of yeast strains Y0139 (Ask1-V5-LexA) and Y0045 (control strain). Genomic DNA samples from G1-arrest 24 min release and 60 min + HU release were analyzed by qPCR. Bar plots show copy-number ratios of ARS313 to the late-replicating region (Chr 4) with error bars indicating standard deviation from n = 3 biological replicates,^∗^p < 0.05, unpaired t test. (D) Replication profiles of chromosome III at 24 min after release into S-phase in the same strains as in (C). (E) Replication profiles of chromosome III at 60 min release into S-phase and 200 mM HU treatment in the same strains as in (C). (F) Experimental outline for tethering Ask1 at ARS305. Yeast strain Y0138 expresses Ask1 as LexA-V5 fusion protein for artificial recruitment of Ask1 to ARS305. (G) ChIP-qPCR analysis using V5-antibodies to immunoprecipitate Ask1-V5-LexA at the indicated genomic regions (n = 1 biological replicate). (H) RT analysis in yeast strains Y0138 (Ask1-V5-LexA) and Y0016 (control strain). Genomic DNA samples from G1-arrest, 24 min release and 60 min + HU release were analyzed by qPCR. Bar plots show copy-number ratios of ARS305 to late-replicating region (Chr 4) with error bars indicating standard deviation from n = 3 biological replicates,^∗^p < 0.05, unpaired t test. (I) Replication profiles of chromosome III at 24 min after release into S-phase in the same strains as in (H). (J) Replication profiles of chromosome III at 60 min release into S-phase and 200 mM HU treatment in the same strains as in (H). See also Figure S9.

Consequently, we asked if and how artificial tethering of Ask1 at other EE or LI locations can affect chromosome III replication. To this end, we took advantage of our LEXA strains and recruited Ask1-V5-LexA to ARS313 (Figures 4A–4E) or ARS305 (Figures 4F–4J). ChIP-qPCR with V5 antibodies confirmed similar enrichment of Ask1-V5-LexA in vicinity of the LEXA binding sites of ARS313 (Figure 4B) and ARS305 (Figure 4G), indicating successful recruitment of Ask1 in both experiments. Intriguingly, Ask1 recruitment to ARS313 advanced replication of ARS313 LI origin as shown by both qPCR and DNA-sequencing analysis (Figures 4C, 4D, and 4E, black arrow). In addition, neighboring active replication origins ARS307, ARS308, and ARS309 showed increased activity (Figure 4D, green asterisks), whereas intervening LI regions showed decreased DNA copy number similar to the effect observed after Ask1 tethering to ARS316 (Figure 4D, red asterisk). Thus, Ask1 recruitment to ARS313 positively affected a cluster of EE origins in close neighborhood of the targeted origin.

Tethering Ask1 to ARS305 on the left chromosome arm gave similar results. First, DNA sequencing analysis showed a small increase in ARS305 origin activity at the 24 min time point (Figures 4I and 4J). Second, the same cluster of origins (ARS307, ARS308, and ARS309) advanced replication to a similar extent as after recruitment to ARS313 (Figure 4I, green asterisks). Under 60 min + HU conditions, Ask1 recruitment to ARS305 did not show dramatic changes in the firing of EE origins except for a region around ARS308, but further dropped copy-number ratios of several late-replicating inter-origin regions (Figure 4J, red asterisks). In general, replication profiles in all tethering and control strains showed very high correlations (Figure S9B), except for one region with high variability on the right arm of chromosome III (Figure S9C, black arrow). Importantly, all changes reported above were highly reproducible and significant across three biological replicates, thereby avoiding such rare regions with large intrinsic noise in RT. Together, these results suggested a model where the Ask1/DASH complex could provide individual chromosomal attachment points that support efficient origin clustering in G1-phase and therefore explain the observed long-range effects of neighboring chromosomal domains.

### Targeted degradation of the DASH complex subunit Ask1 changes RT of specific chromosomal domains

**Figure 5:**
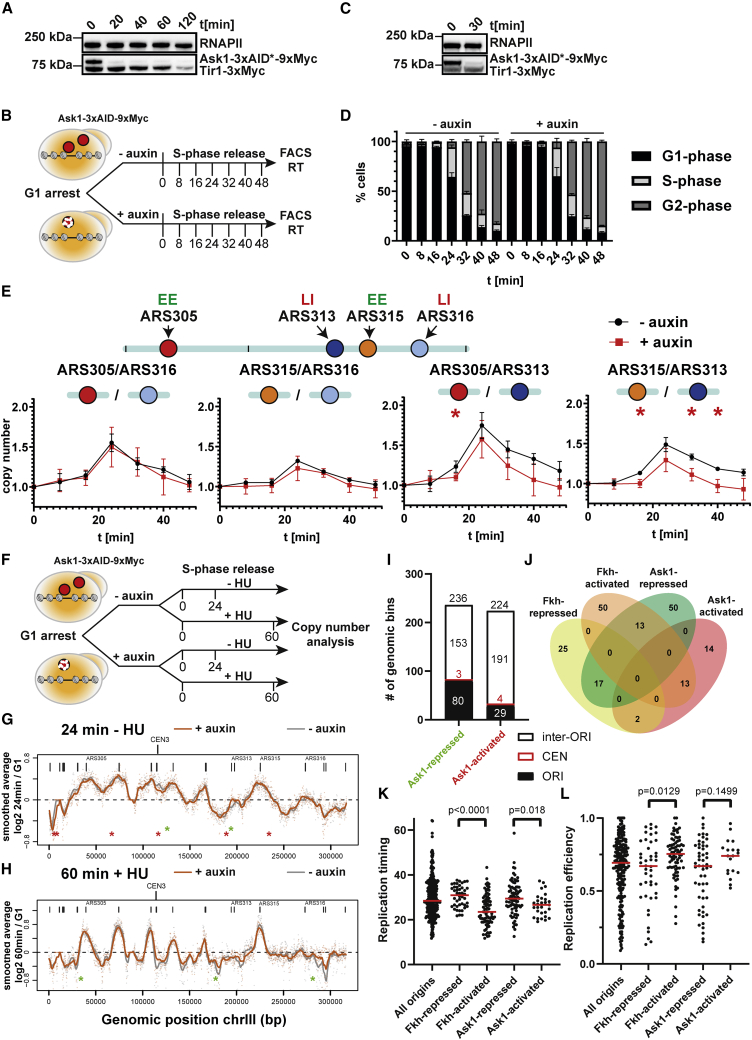
Targeted degradation of Ask1 reveals Ask1-dependent origins (A) Yeast strain Y0123 expressing Ask1 with 3xAID^∗^-9xMyc tag was arrested in G1-phase in the presence and absence of 1 mM auxin for indicated time points. Western blot analysis shows rapid degradation of Ask1 after 20–40 min. 3xMyc-tagged Tir1 and RNAPII were used as loading controls (n = 1 biological replicate). (B) Schematic outline of G1-arrest and release. Y0123 was grown to logarithmic phase, arrested in G1-phase, and cultured for 30 min in the presence or absence of 1 mM auxin before release into S-phase. Samples were taken at the indicated time points for FACS and RT analysis. (C) Western blot shows rapid degradation of Ask1 after 30 min of auxin incubation during G1-arrest (n = 1 biological replicate). (D) S-phase progression analysis by flow cytometry. Total DNA content was measured by SYTOX green staining. Bar graphs show percentages of G1-, S-, and G2/M-phase cells at the indicated time points. Bar plots with error bars indicating standard deviation from n = 3 biological replicates. (E) Samples for genomic DNA extraction were taken at the indicated time points for qPCR analysis. Plots show copy-number ratios of early (ARS305 and ARS315) to late origins (ARS313 and ARS316) with error bars indicating standard deviation from n = 3 biological replicates,^∗^p < 0.05, unpaired t test. (F) Schematic outline of G1-arrest and release. Y0123 was grown to logarithmic phase, arrested in G1-phase, and then cultured for 30 min in the presence or absence of 1 mM auxin. Both cultures were released into S-phase and harvested 24 min after release or 60 min after release in the presence of 200 mM HU. (G) Replication profiles of chromosome III at 24 min after release into S-phase with and without addition of auxin in Y0123. (H) Replication profiles of chromosome III at 60 min release into S-phase in the presence of 200 mM HU with and without addition of auxin in Y0123. (I) Bar graph of the number of genomic bins that advanced (Ask1-repressed) or delayed RT (Ask1-activated) in proximity (within < 5 kb) to centromeres (CEN), origins (ORI), or inter-origin locations. (J) Venn diagram of Ask1-activated, Ask1-repressed, Fkh-activated, and Fkh-repressed origin classes. (K) RT distribution from Raghuraman et al.^35^ of all origins, Fkh1-activated, Fkh-repressed, Ask1-activated, and Ask1-repressed origin classes (p values from unpaired t test). (L) Replication efficiency distribution^14^ of all origins, Fkh1-activated, Fkh-repressed, Ask1-activated, and Ask1-repressed origin classes (p values from unpaired t test). See also Figures S9 and S10.

To further test the functional relevance of Ask1/DASH on RT, we next wanted to study the effect of global Ask1 protein loss on DNA replication. Because ASK1 is essential, we took advantage of an auxin-inducible degron (AID) system to conditionally degrade AID-tagged Ask1 protein.^52^ After 20–40 min of auxin treatment, Ask1 protein levels were undetectable in the Ask1-3xAID^∗^-9xMYC strain (Figure 5A), which provided us with a tool to study the role of Ask1 in DNA replication. We arrested cells in G1-phase, followed by Ask1 degradation for 30 min and subsequent release into S-phase (Figure 5B). Ask1 degradation was confirmed by western blot (Figure 5C). In time course experiments, we monitored S-phase progression using flow cytometry. We could not detect a major difference in S-phase progression between control and Ask1-depleted cells (Figure 5D), suggesting that Ask1 does not perturb DNA replication on a global scale.

However, excess origins may compensate acute loss of Ask1 to complete DNA synthesis and therefore mask changes in RT at specific Ask1-regulated origins. Therefore, we determined the effect of Ask1 degradation on RT of specific EE or LI origins using DNA copy-number analysis as above (Figure 5E). Ask1-depleted cells (+auxin) showed no significant change in RT of the EE origin ARS305 or ARS315 in comparison with the LI origin ARS316 (Figure 5E, left panels). Importantly, however, the LI origin ARS313 changed significantly compared with EE origins ARS305 and ARS315 between control and Ask1-depleted cells (Figure 5E, right panels). For both origins, Ask1-depleted cells showed lower copy-number ratios, suggesting that replication of ARS313 was more efficient in the absence of Ask1. Similar results were obtained when comparing the RT of the four origins with an independent late-replicating region (Chr4), where only ARS313 revealed significant copy-number changes (Figures S9D and S9E). We conclude that endogenous Ask1 has a functional role in RT at the LI origin ARS313.

To confirm these results and extend the analysis to the entire yeast genome, we next monitored DNA replication in Ask1 wild-type (–auxin) and Ask1-depleted cells (+auxin) by DNA sequencing (Figure 5F). After release into S-phase for 24 min, we observed three major changes in the replication profile of chromosome III. As expected, a ∼15 kb region around ARS313 advanced replication (Figure 5G, green asterisk), confirming our qPCR results (Figure 5F). Interestingly, we observed one additional region on chromosome III that shifted to earlier replication in the vicinity of ARS309. At the same time, several regions showed delayed replication in Ask1-depleted cells, including the left end of the chromosome upstream of ARS305 as well as a region around ARS308, which overlaps with the centromere of chromosome III (CEN3) (Figure 5G, red asterisks). In cells released for 60 min + HU treatment, the general pattern of EE origin firing was preserved with only very few regions showing small but significant changes toward earlier replication (Figure 5H, green asterisks). Importantly, ARS313 and CEN3 were not significantly affected in this condition, suggesting that Ask1-dependent RT changes do not occur at the initiation stage of early origins, but rather at a later elongation stage during S-phase progression (Figure 5H). These data suggest that chromosome III normally contains three major positions where Ask1/DASH exerts a functional role in RT, including ARS313, ARS309, and the centromeric ARS308.

Importantly, we obtained similar results for all chromosomes, where 236 regions advanced in Ask1-depleted cells (Ask1 repressed) (Figures 5I, S9F, and S10, green asterisks), whereas 224 regions showed delayed replication compared with Ask1 wild-type cells (Ask1 activated) (Figures 5I, S9F, and S10, red asterisks). Among these regions, centromeres of chromosomes V, VII, and VIII (CEN5, CEN7, and CEN8) showed similar replication delays as CEN3, whereas three other chromosomes advanced centromeric replication (CEN2, CEN11, and CEN15) in Ask1-depleted cells. In general, regions with delayed replication were more likely located in inter-origin regions as only 29 of 224 regions (13%) overlapped within 5 kb of annotated replication origins. In contrast, regions that advanced replication were more frequently associated with replication origins (80 of 236, 34%) (Figure 5I; Table S4). Consistent with FACS (Figure 5D), these data support the notion that endogenous Ask1/DASH recruitment at origins does not act as a global regulator of RT, but rather regulates replication of specific chromosomal regions, including a subset of origins, centromeric as well as non-origin regions. The fact that the changes were often scattered throughout individual chromosomes with both positive and negative effects suggested that the function of Ask1/DASH in RT may not be locally restricted to individual origins but rather involve concerted changes of larger chromosomal domains.

Previous studies showed that efficient origin clustering in the nuclear center is important for early RT, whereas late-replicating regions are preferentially located at the nuclear periphery.^53^^,^^54^ Mechanistically, it was shown that forkhead transcription factors Fkh1/Fkh2 are required for this early origin clustering.^49^^,^^54^^,^^55^^,^^56^ When comparing Fkh-regulated origins with Ask1-regulated origins, we found that out of 80 Ask1-repressed origins, only 17 overlapped with Fkh-repressed origins and 13 overlapped with Fkh-activated origins. Similarly, out of 29 Ask1-activated origins, only 13 overlapped with Fkh-activated origins and 2 overlapped with Fkh-repressed origins (Figure 5J). This suggested that Ask1-regulated origins are distinct from Fkh-regulated origins. Intriguingly, however, Ask1-dependent origins showed similar replication timing and efficiency properties as Fkh-dependent origins, namely that activated origins show earlier RT and higher replication efficiency than repressed origins (Figures 5L and 5K). This indicates that Ask1 and Fkh recruitment to origins have a similar functional impact on origins, suggesting a mechanistic link of Ask1 with efficient origin clustering in G1-phase as reported previously for Fkh-regulated origins.

### Disrupting microtubule dynamics in G1-phase interferes with Ask1-dependent LI origin ARS313

**Figure 6:**
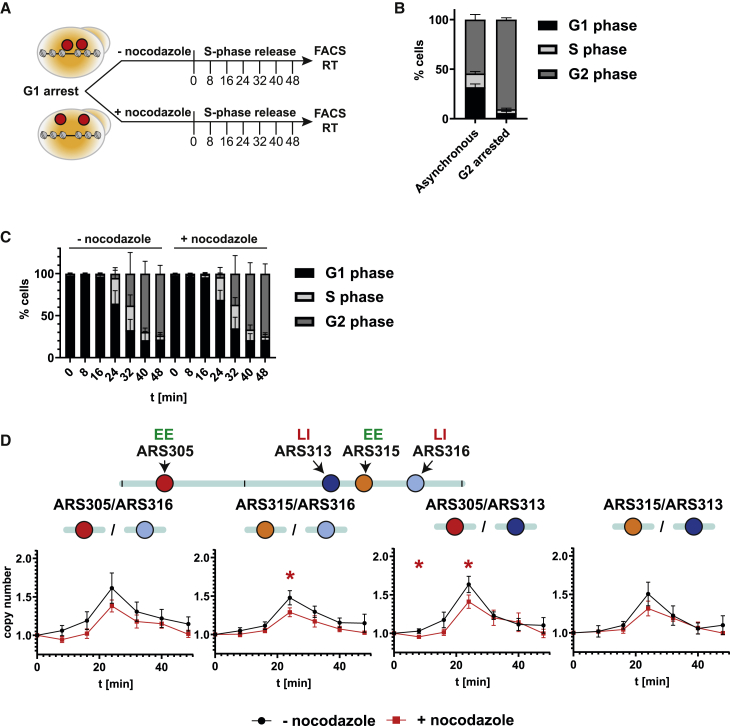
Disrupting microtubule dynamics interferes with RT of Ask1-dependent origins (A) Schematic outline of G1-arrest and release. Y0001 was grown to logarithmic phase, arrested in G1-phase, and then cultured in the presence or absence of 15 μg/mL nocodazole before release into S-phase. Samples were taken at the indicated time points for FACS and RT analysis. (B) Flow cytometry analysis of an asynchronous yeast culture (Y0001) with or without treatment with 15 μg/mL nocodazole for 2 h. Bar graphs depict percentage of G1-, S-, and G2-phase cells at the indicated time points. Bar plots with error bars indicating standard deviation from n = 3 biological replicates. (C) S-phase progression analysis by flow cytometry. Total DNA content was measured by SYTOX green staining. Bar graphs depict percentage of G1-, S-, and G2-phase cells at the indicated time points with error bars indicating standard deviation from n = 3 biological replicates. (D) Samples for genomic DNA extraction were taken at the indicated time points for qPCR copy number analysis. Plots show average copy number ratios of early (ARS305 and ARS315) to late origins (ARS313 and ARS316) with error bars indicating standard deviation from n = 3 biological replicates,^∗^p < 0.05, unpaired t test.

As the canonical function of Ask1/DASH is to form microtubule-encircling rings to allow efficient attachment of microtubules to yeast kinetochores,^44^^,^^45^^,^^46^ we hypothesized that Ask1/DASH binding to selected non-centromeric regions may serve as specific attachment points to connect ends of microtubules with chromatin and therefore provide a structural framework for intranuclear organization of chromosomes. To test this hypothesis, we treated G1-phase arrested cells with nocodazole for 2 h to inhibit microtubule dynamics. Cells were released into S-phase to determine the RT of Ask1-dependent origins on chromosome III (Figure 6A). Addition of nocodazole to asynchronously growing cells efficiently arrested them in G2-phase (Figure 6B), showing that the time and used concentration of nocodazole was sufficient to block microtubule dynamics. In time course experiments, we monitored S-phase progression using FACS. Like results obtained after auxin-mediated Ask1 degradation (Figure 5D), we could not detect major differences in S-phase progression between control and nocodazole-treated cells (Figure 6C), suggesting that inhibition of microtubule dynamics does not perturb replication on a global scale.

If the mechanistic role of Ask1/DASH is to allow microtubule binding and facilitate origin clustering/chromosome organization, our results would predict that nocodazole-induced changes in RT would phenocopy results obtained from RT analysis in Ask1-depleted cells. Therefore, we determined replication profiles of EE and LI origins on chromosome III in the absence or presence of nocodazole (Figure 6D). Nocodazole-treated cells showed slightly lower copy-number ratios when comparing both EE origins ARS305 and ARS315 with LI origins ARS313 or ARS316. Importantly, the EE origin ARS305 showed most significant changes in RT in comparison with the LI origin ARS313, consistent with the RT changes in Ask1-depleted cells where these two regions most prominently shifted RT in opposite directions (Figures 5E and 5G). We conclude that Ask1/DASH has a functional role at selected chromosomal origins and this role is dependent on functional microtubules. Therefore, our data show a previously not established connection of the RT program and intact microtubule dynamics.

## Discussion

### Single-locus proteomics analysis of EE and LI origins

Identifying factors at specific target genomic region in an unbiased manner remains a major challenge in chromatin research. In this study, we developed an efficient system to specifically excise and purify distinct replication origins from yeast chromosome III. Purity and yield of our single-step purifications overcomes many existing limitations of locus-specific chromatin purification methods,^57^ allowing us to report a comprehensive proteomic analysis of selected EE and LI origins. Importantly, these domains were previously located in their native chromosomal context, whereas other approaches to purify origin chromatin reported so far were restricted to the single-copy EE ARS1 origin cloned on plasmids,^20^ which might not fully reflect the chromatin context of endogenous chromosomes.

Our site-specific recombination approach necessitates genetic modifications, and we cannot exclude the possibility that binding of LexA-TAP to origin-proximal LEXA sites or the process of recombination may trigger eviction or shifts of individual chromatin components. However, the RTs of the origins in the genetically modified cells were identical to wild-type cells and exactly as expected for EE and LI origins (Figure S4). Furthermore, ChIP analysis against histones and MCMs in the endogenous chromosomal context validated MS results regarding MCM:histone stoichiometry (Figures S7A and S7B). Thus, we believe that critical chromatin features defining different RT profiles were not affected by our manipulations. Nevertheless, maintaining the stoichiometry of all locus-bound factors during biochemical purification is a major challenge. Our choice to purify origins under native conditions without assistance from chemical crosslinkers to stabilize more transient chromatin interactions is a clear limitation that may explain why some expected interactions were not retrieved in our analysis. For example, we failed to identify Fkh1/Fkh2 at ARS305, a well-established forkhead-activated origin,^54^^,^^58^ and we could also not recover ORC subunits in three of four origin purifications. Therefore, we cannot claim to recover a “complete” list of all origin-bound proteins, which may explain the surprisingly limited overlap of factors between the two classes of EE and LI origins (Figure 2).

Another surprising result was that we purified about ∼10-fold more protein factors from ARS313 compared with every other origin (Figure 2), even though all purified domains have similar sizes and purification efficiencies (Figures 1E–1G). Lowering the salt concentration gave identical results (Figures S8C and S8E), suggesting that this effect may be biologically relevant. Further bioinformatic profiling of chromatin around the four targeted origins did not reveal striking differences of ARS313 to the other origins (Figure S6), although we note that ARS313 was largely devoid of H3K4me3 and characterized by comparably lower levels of H3K9ac and H4K12ac. Thus, the origin is potentially located in a transcriptionally less active and more condensed chromatin region. As higher levels of chromatin condensation can stabilize chromatin binding and reduce dissociation of chromatin-binding factors,^59^ this could be a reasonable explanation for this imbalanced protein recovery at ARS313. Nevertheless, we cannot exclude the possibility that intrinsic chromatin features, such as specific combination of histone modifications at ARS313 renders this domain in a more “sticky” state that could artificially increase protein association during the purification. One solution is to use chemical crosslinkers that allow purification under more stringent conditions. Future work using optimized crosslinking conditions or crosslinking reagents might thus help to capture transient, but biologically important proteins with this system.

### The DASH complex subunit Ask1 is a *bona fide* RT factor

One of the unexpected findings was that several Ask1/DASH complex subunits co-purified with ARS305 and ARS313. Degrading Ask1 in G1-phase had no measurable impact on global S-phase progression (Figure 5D), suggesting that Ask1 does not perturb replication on a global scale. However, RT measurement of individual origins along chromosome III revealed a functional role of Ask1 in RT of ARS313 and other origins tested on chromosome III (Figures 5E–5H).

How might Ask1/DASH mechanistically advance or delay RT of specific replication origins? In mitosis, this complex assembles into a microtubule-encircling ring that helps attaching microtubules to the outer kinetochore. DASH complex-mediated attachment of microtubules at origins in G1-phase may serve a similar function to establish correct sub-nuclear positioning of chromosomes. Indeed, it was shown that Fkh transcription factors are required for clustering early origins and their emergence in replication factories.^54^ Thus, in addition to centromere and telomere anchoring at the nuclear membrane, selected attachment of microtubules via Ask1/DASH could provide the structural framework for sub-nuclear rearrangement of specific origins and explain how this movement of individual genomic loci is physically achieved. This is consistent with our finding that Ask1-activated and Ask1-repressed replication origins show comparable replication features as Fkh-activated and Fkh-repressed replication origins (Figures 5K and5L). A function of Ask1/DASH in 3D organization of specific origins would also explain the surprising result that both tethering (Figures 3 and 4) and degrading (Figure 5) Ask1 advances replication of ARS313. In all tethering experiments of Ask1, we observed a consistent and reproducible advancement of RT of the Ask1-recruited origin (Figures 3 and 4), suggesting that the presence of Ask1/DASH is generally supportive of efficient origin replication. One key difference between Ask1-tethering and degradation experiments is that tethering of Ask1 is locally restricted to one chromosomal location containing LEXA-binding sites, whereas Ask1 degradation occurs globally and can therefore affect multiple sites per chromosome. As result, multiple chromosome regions can be rearranged in a likely complex way. Consistently, Ask1 degradation caused multiple other changes of RT along chromosome III including the CEN3 region and inter-origin regions (Figure 5G). Together, the overall rearrangement of chromosome III after Ask1 degradation may lead to more central nuclear positioning of ARS313, adjusting its replication profile to other early origin clusters. However, to address this point concisely, a more detailed chromosome conformation analysis such as Micro-C^60^ would be necessary. In addition, the mechanism by which the local chromatin of origins impacts the selective recruitment of Ask1/DASH at specific origins and how Fkh1/Fkh2 might interplay and regulate this directionality require future investigations. Overall, our origin purification system of selected EE and LI origins provide an ideal framework to understand the basis for differential origin regulation and its connection to chromosomal domain organization.

### Limitations of the study

Initial ChIP-qPCR experiments to verify direct interaction of endogenous Ask1/DASH with origins suffered from low IP efficiencies, which is likely caused by the fact that Ask1/DASH has no direct DNA-binding domain for efficient crosslinking. Thus, genome-wide binding profiles of Ask1/DASH to determine physical association of Ask1 with origins remains to be investigated.

As all DASH complex subunits are essential and the tagging of other subunits did not yield viable clones, we were unable to experimentally address the question whether this function is specific to Ask1 alone or whether other complex subunits have similar effects. However, based on the structure and canonical function of DASH to encircle microtubules and our finding that microtubule dynamics are important for RT changes of Ask1-dependent origins (Figure 6), we favor a model where the whole complex is required to perform this cellular function.

## STAR★Methods

### Key resources table

**Table:** 

REAGENT or RESOURCE	SOURCE	IDENTIFIER
**Antibodies**		

Rabbit Peroxidase Anti-Peroxidase Soluble Complex antibody	Sigma-Aldrich	Cat# P1291//RRID:AB_1079562
Mouse Monoclonal anti-V5	ThermoFisher	Cat# R96025//RRID:AB_159313
Mouse Monoclonal anti-c-MYC clone 9E10	Sigma-Aldrich	Cat# 11667149001//RRID:AB_390912
Rabbit Monoclonal anti-RNA polymerase II CTD repeat YSPTSPS (phospho S2)	Abcam	Cat# ab193468//RRID:AB_2905557
Rabbit monoclonal anti-H2A (phospho S129)	Abcam	Cat# ab181447//RRID: N/A
Rat monoclonal anti-HA (3F10)	Sigma-Aldrich	Cat# 11867423001//RRID:AB_390918
Rabbit polyclonal anti-H3	Abcam	Cat# ab1791//RRID:AB_302613
HRP-conjugated goat anti-rabbit	Invitrogen	Cat# G21234//RRID:AB_2536530
HRP-conjugated goat anti-mouse	Invitrogen	Cat# G21040//RRID:AB_2536527

**Chemicals, peptides, and recombinant proteins**		

Yeast Mating Factor Alpha	Biomol	Cat# Y2016.5
Hydroxyurea	Biomol	Cat# H9120.10
Protease and Phosphatase Inhibitor Cocktail 100x	ThermoFisher	Cat# 78446
Trichostatin A	Sigma-Aldrich	Cat# T8552
Sirtinol	TargetMol	Cat# T6671
Rabbit IgGs	Sigma-Aldrich	Cat# I5006
Proteinase K	Serva	Cat# 33756
RNAse A	ThermoFisher	Cat# EN0531
Formaldehyde	ThermoFisher	Cat# 28908
Pronase	Sigma-Aldrich	Cat# 53702
Nocodazole	Merck	Cat# 487928
Auxin	MP Biomedicals	Cat# 102037
Zymolyase	Biomol	Cat# Z1005
Sytox Green	ThermoFisher	Cat# S7020
Lysyl Endopeptidase	Wako Chemicals	Cat# 121-05063
Trypsin	Promega	Cat# V528A

**Critical commercial assays**		

NEBNext® Ultra™ II DNA Library Prep with Sample Purification Beads	NEB	Cat# E7103
RadPrime DNA Labeling System	ThermoFisher	Cat# 18428011

**Deposited data**		

Mass spectrometry Data	This paper	PRIDE: PXD031984
DNA copy number sequencing	This paper	GEO: GSE212974

**Experimental models: Organisms/strains**		

Yeast strains	This paper (Table S4)	N/A

**Oligonucleotides**		

	This paper (Table S4)	N/A

**Recombinant DNA**		

Plasmids	This paper (Table S4)	N/A

**Software and algorithms**		

Proteome Discoverer v2.5	ThermoFisher	N/A
ImageJ	NIH	https://imagej.net/ij/index.html
FlowJo v10	BD Bioscience	
trim-galore v0.6.7	https://doi.org/10.5281/zenodo.5127899	https://www.bioinformatics.babraham.ac.uk/projects/trim_galore/
bowtie2 v2.4.5	https://doi.org/10.1038/nmeth.1923	https://bowtie-bio.sourceforge.net/bowtie2/index.shtml
Samtool v1.14	https://doi.org/10.1093/gigascience/giab008	https://www.htslib.org/doc/samtools.html
R v4.1.2	R Core Team	https://www.r-project.org
GenomicAlignments v1.30.3	https://doi.org/10.1371/journal.pcbi.1003118	https://bioconductor.org/packages/release/bioc/html/GenomicAlignments.html
GenomicRanges v1.46.1	https://doi.org/10.1371/journal.pcbi.1003118	https://bioconductor.org/packages/release/bioc/html/GenomicRanges.html
Zoo package v1.8-10	N/A	https://cran.r-project.org/web/packages/zoo/index.html
Snakemake.minimal v5.2.4	https://doi.org/10.12688/f1000research.29032.2	https://snakemake.readthedocs.io/en/stable/index.html

### Resource availability

#### Lead contact

Further information and requests for resources and reagents should be directed to the lead contact, Stephan Hamperl (stephan.hamperl@helmholtz-muenchen.de).

#### Materials availability

This study did not generate any unique reagents.

### Experimental model and subject details

Unless noted otherwise, standard molecular biology techniques were used for cloning of plasmids and lithium acetate transformation of yeast cells. All *S. cerevisiae* strains used in this study are based on Y0001 (wild-type). Complete lists of oligonucleotides, plasmids and yeast strains with genotypes can be found in Table S4. Yeast strains and plasmids are available upon request.

### Method details

#### Yeast cell culture

Yeast cells competent for recombination were cultured in YPR medium at 30°C to an OD600 of 1.0. Cells were then simultaneously arrested in G1 phase by addition of alpha factor (50 ng/mL) and expression of R recombinase induced by addition of galactose to a final concentration of 2% (w/v). Cells were grown for an additional 2h at 30°C before harvesting by centrifugation (10min, 7.000 g at 4°C), yielding approximately 1.5g of yeast cells wet weight per liter of medium. Cells were resuspended with water, before being pelleted in sealed 25mL syringes by centrifugation (10min, 7.000 g at 4°C). The supernatant was decanted, the syringe unsealed and the cells were extruded into liquid nitrogen. The resulting cell “spaghetti” can be stored at −80°C until further usage.

#### Spot tests

Sensitivity of temperature-sensitive yeast strains to hydroxyurea (HU) was determined by spotting serial dilutions of exponentially growing yeast cultures on YPD plates with or without 10mM hydroxyurea and incubation at the indicated temperatures.

#### Affinity purification of chromatin domains

A commercial coffee grinder (Gastroback, 42601) was pre-cooled by grinding 30–50g of dry ice twice. The resulting powder of dry ice was discarded. 3g of frozen cells were mixed with ∼90g of dry ice in the coffee mill. Grinding was repeated ten times for 30sec with 30sec breaks to prevent overheating of the coffee mill. Shaking of the coffee mill while grinding prevented the dry ice–cell powder from sticking to the inside wall of the grinding chamber. The fine powder of ground yeast can be stored at −80°C. After evaporation of dry ice, the powder was dissolved in 0.75mL of cold buffer MB200 [20mM Tris–HCl (pH 8), 200mM KCl, 5mM MgAc, 0.5% Triton X-100, 0.1% Tween 20, 1mM DTT] or buffer MB150 [20mM Tris–HCl (pH 8), 150mM KCl, 5mM MgAc, 0.5% Triton X-100, 0.1% Tween 20, 1mM DTT], both supplied with 1× protease and phosphatase inhibitors (Protease and Phosphatase Inhibitor Cocktail 100x, Thermo Fisher Scientific) and 1x histone deacetylase inhibitors (0.5μM Trichostatin A, 25μM Sirtinol), per 1g of ground yeast cells. The respective MB200 or MB150 buffer was then used throughout the complete purification. The cell lysate was cleared from cell debris by centrifugation with 16.000g for 30 min at 4°C. To generate the affinity resin, rabbit IgGs (Sigma) were added to epoxy-activated magnetic beads (BcMag, Bioclone Inc.) in a ratio of 0.17mg IgGs/mg of beads according to a published protocol.^34^ The IgGs coupled to magnetic beads were equilibrated with buffer MB with 1× protease and phophatase inhibitors (Protease and Phosphatase inhibitor Cocktail 100x, Thermo Fisher Scientific) and 1x histone deacetylase inhibitors (0.5μM Trichostatin A, 25μM Sirtinol) before use. For the purification of the chromatin rings 333μL of magnetic bead slurry with coupled IgGs were added to the cell lysate. The cell lysate-bead suspension was incubated on a rotating wheel for 2h at 4°C. Beads were washed three times with 750μL of cold buffer MB with 1× Protease and Phophatase inhibitors (Protease and Phosphatase Inhibitor Cocktail 100x, Thermo Fisher Scientific) and 1x histone deacetylase inhibitors (0.5μM Trichostatin A, 25μM Sirtinol). Between each washing step, the beads were gently rotated for 5min. Finally, the beads were washed twice with 750μL of cold buffer AC (100mM NH_4_Ac pH 7.4 titrated with 2M NH_3_, 0.1mM MgCl_2_). Chromatin rings were eluted by adding 500μL 0.5M NH_4_OH, thorough mixing and incubating at room temperature for 30min. This process was repeated once and both eluates were combined to a final volume of 1mL and frozen at −80°C before submission to mass spectrometry.

#### DNA analysis of the purification fractions

H_2_O was added to the DNA samples taken during the purification process to a final volume of 100μL. 1ng of plasmid K71 was also added to every sample as a spike-in to normalize for different DNA extraction efficiencies. 100μL of IRN buffer (50mM Tris-HCl pH8, 20mM EDTA, 0.5M NaCl, 0.5% SDS, 10μL Proteinase K (10 mg/mL) were added together with 1μL of RNAse A (10 mg/mL), followed by a 1h incubation step at 37°C. Subsequently, 200μL Phenol:Chloroform:Isoamyl Alcohol (25:24:1, v/v) was added, followed by 2 × 10sec thorough vortexing. The solution was centrifuged for 5 min at 16.000g. The supernatant was transferred to a fresh 1.5mL tube containing 600μL of ethanol and 1.5μL glycogen (10 mg/mL). The tube was left at −20°C overnight. Next, the solution was centrifuged with 16.000 g at 4°C for 30min. The supernatant was discarded and 150μL of 70% ethanol was added to the pellet. After another centrifugation step with 16.000 g at 4°C for 10min, the supernatant was discarded and the DNA pellet dried at room temperature for 10min. The dried pellet was then resuspended in 50μL H_2_O. For further analysis, a restriction digestion was performed to analyze the DNA samples in subsequent qPCR reactions. The restriction enzymes used for linearizing the circular DNA were HpaI (ARS305+/−3), BbsI (ARS313+/−3), NcoI (ARS315+/−3) and HpaI (ARS316+/−3). qPCR analysis was performed using the following primer pairs: ARS305: 0463/0466; ARS313: 0552/0553; ARS315: 0970/0971; ARS316: 0837/0838. Primers 0137 and 0138 are used to detect the K71 spike-in and primers 0301 and 0302 were used to detect the unrelated genomic PDC1 locus.

#### Filter aided sample preparation (FASP) digest of protein samples for mass spectrometry

The eluates from the purified samples were dried using a speed vac vacuum concentrator and resolved in 300μL 50mM ammonium bicarbonate (ABC) and digested using a modified FASP procedure.^61^^,^^62^ After protein reduction and alkylation using DTT and IAA, the proteins were centrifuged on a 30kDa cutoff filter device (Sartorius), washed thrice with UA buffer (8M urea in 0.1M Tris/HCl pH 8.5) and twice with 50mM ABC. The proteins were digested for 2h at room temperature using 0.5μg Lys-C (Wako Chemicals, Neuss, Germany) and for 16 h at 37°C using 1μg trypsin (Promega, Mannheim, Germany). After centrifugation (10 min at 14.000 g) the eluted peptides were acidified with 0.5% TFA and stored at −20°C.

#### LC-MS/MS measurements and quantitative data analysis using progenesis QI for proteomics

LC-MS/MS analysis was performed on a Q-Exactive HF and HF-X mass spectrometer (Thermo Fisher Scientific) online coupled to an Ultimate 3000 nano-RSLC (Thermo Fisher Scientific). Tryptic peptides were automatically loaded on a C18 trap column (300 μm inner diameter (ID) × 5 mm, Acclaim PepMap100 C18, 5 μm, 100 Å, LC Packings) at 30 μL/min flow rate prior to C18 reversed phase chromatography on the analytical column (nanoEase MZ HSS T3 Column, 100Å, 1.8μm, 75μm × 250mm, Waters) at 250 nL/min flow rate in a 95 min non-linear acetonitrile gradient from 3 to 40% in 0.1% formic acid. Profile precursor spectra from 300 to 1500 m/z were recorded at 60,000 resolution with an automatic gain control (AGC) target of 3e6 and a maximum injection time of 30ms and 50ms. TOP10 and TOP15 fragment spectra of charges 2 to 7 were recorded at 15,000 resolution with an AGC target of 1e5, a maximum injection time of 50ms, an isolation window of 1.6 m/z, a normalized collision energy of 28 and a dynamic exclusion of 30 s.

#### Protein identification and label-free quantification

Proteome Discoverer 2.5 software (Thermo Fisher Scientific; version 2.5.0.400) was used for peptide and protein identification via a database search (Sequest HT search engine) against Swissprot yeast database (Release 2017_04, 6721 sequences), considering full tryptic specificity, allowing for up to two missed tryptic cleavage sites, precursor mass tolerance 10 ppm, fragment mass tolerance 0.02Da. Carbamidomethylation of Cys was set as a static modification. Dynamic modifications included deamidation of Asn, Gln and Arg, oxidation of Pro and Met; and a combination of Met loss with acetylation on protein N-terminus. Percolator was used for validating peptide spectrum matches and peptides, accepting only the top-scoring hit for each spectrum, and satisfying the cutoff values for FDR <5%, and posterior error probability <0.01. The final list of proteins complied with the strict parsimony principle. The quantification of proteins was based on abundance values for unique peptides. Abundance values were normalized on total peptide amount to account for sample loading errors. The protein abundances were calculated summing up the abundance values for admissible peptides. The final protein ratio was calculated using median abundance values of three replicate analyses each. The statistical significance of the ratio change was ascertained employing the T test approach described in^63^ which is based on the presumption that we look for expression changes for proteins that are just a few in comparison to the number of total proteins being quantified. The quantification variability of the non-changing “background” proteins can be used to infer which proteins change their expression in a statistically significant manner.

#### Chromatin immunoprecipitation

A 100mL yeast culture was grown to OD600 of 0.6 and then arrested at G1-phase by addition of alpha factor (50 ng/mL) for 2h. 45mL of the culture were transferred to a 50mL falcon tube. For crosslinking, formaldehyde was added to a final concentration of 1%. After 15min shaking at 30°C, the reaction was quenched by the addition of glycine to a final concentration of 128mM. After 5min more shaking at 30°C, cells were pelleted by centrifugation (3.000g, 2 min at 4°C). The cells were washed once with 45mL cold PBS. After pelleting again, cells were resuspended in 1mL cold PBS and then transferred into a 1.5mL tube. After another centrifugation step (16.000g, 1 min at 4°C), the supernatant was discarded and the remaining yeast pellet was frozen in liquid nitrogen for storage at −20°C.

The pellets were washed with 500μL Lysis buffer (50mM HEPES pH 7.5, 140mM NaCl, 5mM EDTA pH8, 5mM EGTA pH8, 1% Triton X-100 (w/v), 0.1% DOC (w/v), 1× protease and phosphatase inhibitors (Protease and Phosphatase Inhibitor Cocktail 100x, Thermo Fisher Scientific) and then resuspended with 500μL Lysis buffer. Precooled glass beads (1mm, BioSpec Products) were added to cover the whole suspension. Cells were disrupted on a VXR basic IKA Vibrax orbital shaker at 2200rpm for three times 15 min at 4°C with 10min breaks on ice in between. To remove the beads from the lysate, the bottom of the 1.5mL tube was pierced using a hot needle and placed into a 15mL falcon tube. After centrifugation (130g, 2 min at 4°C), the beads remained in the 1.5mL tube and the lysate could be collected in the 15mL tube. The volume of the suspension was increased to 1mL with Lysis buffer and transferred to a 1mL Covaris sonication glass tube. Sonification was performed on a Covaris instrument (25min, Peak Incident Power: 140W, Duty Factor: 5%, Cycles/Burst: 200). Afterward, the sheared chromatin was cleared by centrifugation (20min, 16.000 g at 4°C). The supernatant was then transferred to a low-binding 1.5mL tube. The resulting chromatin extract was split into two aliquots. A total of 140μL served as an input control, and 700μL was diluted with 290μL Lysis buffer and incubated for 120 min at 4°C with 40μL of Lysis buffer pre-equilibrated Protein A bead slurry (10001D, Invitrogen) and 10μg of the indicated antibodies.

After immunoprecipitation, the beads were washed three times with lysis buffer, twice with washing buffer I (50mM HEPES pH7.5, 500mM NaCl, 2mM EDTA, 1% [vol/vol] Triton X-100, 0.1% [wt/vol] DOC), and twice with washing buffer II (10mM Tris-HCl pH8.0, 250mM LiCl, 2mM EDTA, 0.5% [vol/vol] Nonidet P-40, 0.5% [wt/vol] DOC), followed by a final washing step with TE buffer (10mM Tris-HCl pH 8.0, 1mM EDTA). A total of 390μL of buffer IRN (50mM Tris–HCl pH8.0, 20mM EDTA, 500mM NaCl) or 250μL was added to the immunoprecipitation (IP) beads and to the input samples, respectively. DNA was isolated by incubation with 10μL RNAse A (10μg/μL) at 37°C. SDS was added to a final concentration of 0.5% together with 10μL Proteinase K (10μg/μL). After incubation for 1h at 56°C, Input and IP samples were decrosslinked at 65°C overnight. DNA was then isolated by phenol-chloroform extraction followed by ethanol precipitation. Both input and IP DNA pellets were suspended in 50μL H2O and analyzed by quantitative PCR using indicated primers.

#### Flow cytometry

500μL samples were taken from yeast cultures (∼OD600 of 0.6). The cells were centrifuged at 3.000g for 2 min at RT. The supernatant was discarded and 1mL 70% Ethanol was added to the pellet. After thorough vortexing, the fixed cell suspensions can be stored at 4°C until further use. 500μL of the suspensions were transferred to a fresh tube and then centrifuged (3.000g, 2min RT). The supernatant was discarded and the pellet dissolved in 300μL 50mM sodium citrate and 0.1 mg/mL RNAse A. After 2h incubation at 50°C, proteinase K was added to a final concentration of 0.1 mg/mL, followed by another 2h incubation at 50°C. 30μL of this sample was then mixed with 170μL 50mM sodium citrate and 0.5μM Sytox Green (S7020, Thermo Fisher Scientific). Prior to FACS analysis, the samples were briefly sonicated (Bioruptor, 5 × 15sec) to detach cell clumps before proceeding with the analysis.

#### Southern blot analysis

Nucleic acids from genomic DNA were separated on a 1% agarose gel and blotted onto a Nylon membrane (Amersham Hybond-N, GE) by capillary transfer in 1M ammonium acetate. DNA probes for hybridization were generated using the RadPrime DNA labeling system (Invitrogen) with incorporation of [α−32P]dATP (Hartmann Analytik) according to the instructions of the manufacturer. Images were acquired with the Typhoon FLA 7000 imaging system.

#### Western blot analysis

2.5 OD600 units of yeast cells were harvested by centrifugation for 5min with 3.000 g at room temperature. Cells were resuspended in 200μL 0.1M NaOH followed by incubation at room temperature for 5min. Whole cell extracts were pelleted for 5min with 3.000 g at room temperature and the pellet was resuspended in 50μL 1x SDS sample buffer. Whole cell extracts were separated by electrophoresis, transferred onto polyvinylidene difluoride membranes (Immobilon®-P PVDF Membrane, Sigma-Aldrich) and blocked in 5% skimmed milk dissolved in 0.05% Tween/PBS for 1h at room temperature. Membranes were incubated with primary antibodies overnight at 4°C followed by washing in 0.1% Tween/TBS. Membranes were incubated with appropriate HRP- linked secondary antibodies at 25°C for 1h and washed thrice prior to signal detection. Membranes were developed by chemiluminescence using ECL reagent.

#### Replication Timing Analysis

A 50mL yeast culture in YPD was grown to OD600 of 0.6 and then arrested in G1-phase by addition of alpha factor (50 ng/mL) for 2h. As indicated, cells were treated with auxin at a concentration of 1mM for 30 min at 30°C to degrade AID-tagged Ask1or with nocodazole at a concentration of 15 μg/mL together with 1%DMSO for 2h at 30°C to destabilize microtubules. To release the cells from the arrest, 125U of Pronase (Sigma- Aldrich, 53702-25KU) and potassium phosphate buffer to a final concentration of 20mM was added. If necessary, 200mM HU was added in the release to induce S phase checkpoint activation. Samples for genomic DNA extraction were taken before the release and every 8min after releasing the cells from the arrest by adding 4.5mL of the culture to 500μL of 1% sodium azide solution (w/v) in 0.2M EDTA. The cells were washed once with water (4.000g, 3 min at 4°C) and the resulting yeast pellets were snapfrozen in liquid nitrogen.

For DNA extraction, the cell pellets were resuspended in buffer RINB (50mM Tris-HCl pH8, 0.1M EDTA, 0.1% (v/v) beta mercaptoethanol). Zymolyase was added to a final concentration of 2% (w/v). After incubating for 1h at 37°C, the solution was supplemented with 1% SDS (w/v), 0.2M NaCl, 0.1 mg/mL RNAse A, and 0.2 mg/mL proteinase K. After incubation for 1h at 55°C, DNA was isolated by phenol-chloroform extraction followed by ethanol precipitation. DNA pellets were suspended in 50μL of H_2_O. 5–10μg of DNA was then digested with EcoRI. The reactions were diluted 1:10 in H_2_O and analyzed by quantitative PCR using primers 0463/0466 (ARS305), 0552/0553 (ARS313), 0970/0971 (ARS315), 0837/0838 (ARS316), and 0834/0835 (ChrVI).

#### DNA copy number sequencing

The DNA samples from the RT experiments were treated an additional time with 0.2 mg/mL RNAse A for 1h at 37°C. A DNA cleanup step was performed using the GeneJET PCR purification Kit (Thermo Scientific). The DNA was eluted in 130μL H_2_O. The whole DNA was added to Covaris sonication tubes (microTUBE AFA Fiber Pre-Slit Snap-Cap 6 × 16mm) for 3min with the following settings: Cycles/burst: 200, intensity: 4, Duty cycle: 10%. Afterward 50μL of the solution was used for the library preparation using the NEBNext ultra II DNA library prep kit following the manufacturer’s protocol. Two subsequent cleanup steps were performed using AMPure SPRI beads to remove remaining adapter dimers. Beads were added in a 1:1 ratio to the library. After a 5min incubation step, the supernatant was removed using a magnetic rack. After two washing steps with 80% EtOH, the beads were dried at room temperature for approximately 2min. The DNA was then eluted from the beads using H_2_O in the desired volume.

### Quantification and statistical analysis

Error bars on Figures 1E, 1F, 1G, 2B, 3B, 3C, 3D, 3F, 4C, 4H, 4E, 5D, 5E, 6B, 6C, 6D, S4A, S4B, S5B, S7A, S7B, S8A, S8B, S8D, S9A and S9E indicate the standard deviation of the biological replicates. All of the statistical details of experiments can be found in the figure legends.

Statistical analysis of the DNA copy number sequencing results in Figures 3G, 3H, 4D, 4E, 4I, 4J, 5G, 5H, and S10 was performed according to the following steps: Paired-end sequencing reads were mapped to the reference genome (Saccharomyces cerevisiae R64-1-1.dna.toplevel.fa) using bowtie2 (version 2.4.5) with the parameters --end-to-end --very-sensitive --no-unal --no-mixed -- no-discordant -I 10 -X 1000. Aligned reads were filtered for mapping quality using samtools (version 1.14) with the parameter -q 12. Read pairs were counted in 500 bp or 1000 bp consecutive genomic windows using R/Bioconductor packages (R version 4.1.2, GenomicAlignments version 1.30.0 and GenomicRanges version 1.46.1). Reads mapped to the mitochondrial genome were excluded from the analysis. Read counts were normalized by the total number of mapped reads and were converted to bedgraph files upon smoothing by the rollmean function (zoo package, version 1.8-10). The log2 ratio between 60 min or 24 min and G1-samples was calculated for each replicate in each condition (i.e. genotype or treatment), respectively. The lowest 5th percentile of the data was filtered out prior log2 transformation. Replicate datapoints were visualized as dot plots, whereas the average of the replicates is shown as a further smoothed curve along the chromosome with a bin size of 500 or 1000bp as indicated. The replicate ‘Y19_24_1’ was removed as outlier. Differential regions were obtained using Welch two sample t test (unequal variances) in each genomic bin with a p value cutoff of 0.05 and a mean difference of at least 0.1. Regions that significantly increased or decreased RT were obtained using Welch two sample t test (unequal variances) in each genomic 500bp bin with a p value cutoff of 0.05 and a mean difference of at least 0.1 and indicated with green or red asterisks, respectively. Plots were generated using R base graphics. All analysis steps were carried out in a reproducible pipeline using snakemake (version snakemake-minimal 5.2.4) and are available upon request.

For the analysis of ChIP-Exo profiles, bigwig files were downloaded from the yeast epigenome project (http://128.84.9.188/YEP_DATA/sampleBigWig/), imported to R (version 4.1.2) using the rtracklayer package (version1.54.0) and converted to coverages using the GenomicRanges package (version 1.46.1). Forward and reverse strand coverages were averaged and smoothed using the zoo package (version 1.8-10). Plots were generated using R base graphics.

## Data Availability

• DNA copy number sequencing data are available on GEO with the accession number GSE212974. The mass spectrometry data have been deposited to the ProteomeXchange Consortium (http://proteomecentral.proteomexchange.org) via the PRIDE partner repository with the dataset identifier PXD031984. • This paper does not report any original code. • Any additional information required to reanalyze the data reported in this paper is available from the lead contact upon request.

## Supplemental information

Document S1. Figures S1–S10

Table S1. DNA sequence features of yeast replication origins, related to Figure 1

Table S2. Complete list of proteins identified at EE and LI replication origins at 200 mM high-salt and 150 mM low-salt conditions, related to Figure 2

Table S3. Complete list of yeast replication origins with their associated RT/efficiency and Fkh/Ask1 status, related Figure 5

Table S4. Complete list of plasmids, yeast strains, and oligonucleotides used in this study, related to STAR Methods

Document S2. Article plus supplemental information

## References

[1] Riera A., Barbon M., Noguchi Y., Reuter L.M., Schneider S., Speck C. (2017). From structure to mechanism—understanding initiation of DNA replication. Genes Dev..

[2] Costa A., Diffley J.F.X. (2022). The initiation of eukaryotic DNA replication. Annu. Rev. Biochem..

[3] Stinchcomb D.T., Struhl K., Davis R.W. (1979). Isolation and characterisation of a yeast chromosomal replicator. Nature.

[4] Theis J.F., Newlon C.S. (1997). The ARS309 chromosomal replicator of Saccharomyces cerevisiae depends on an exceptional ARS consensus sequence. Proc. Natl. Acad. Sci. USA.

[5] Chang F., Theis J.F., Miller J., Nieduszynski C.A., Newlon C.S., Weinreich M. (2008). Analysis of chromosome III replicators reveals an unusual structure for the ARS318 silencer origin and a conserved WTW sequence within the origin recognition complex binding site. Mol. Cell Biol..

[6] Rao H., Stillman B. (1995). The origin recognition complex interacts with a bipartite DNA binding site within yeast replicators. Proc. Natl. Acad. Sci. USA.

[7] Rowley A., Cocker J.H., Harwood J., Diffley J.F. (1995). Initiation complex assembly at budding yeast replication origins begins with the recognition of a bipartite sequence by limiting amounts of the initiator, ORC. EMBO J..

[8] Schmidt J.M., Yang R., Kumar A., Hunker O., Seebacher J., Bleichert F. (2022). A mechanism of origin licensing control through autoinhibition of S. cerevisiae ORC·DNA·Cdc6. Nat. Commun..

[9] Feng X., Noguchi Y., Barbon M., Stillman B., Speck C., Li H. (2021). The structure of ORC-Cdc6 on an origin DNA reveals the mechanism of ORC activation by the replication initiator Cdc6. Nat. Commun..

[10] Wilmes G.M., Bell S.P. (2002). The B2 element of the Saccharomyces cerevisiae ARS1 origin of replication requires specific sequences to facilitate pre-RC formation. Proc. Natl. Acad. Sci. USA.

[11] Zou L., Stillman B. (2000). Assembly of a complex containing Cdc45p, replication protein A, and Mcm2p at replication origins controlled by S-phase cyclin-dependent kinases and Cdc7p-Dbf4p kinase. Mol. Cell Biol..

[12] Ganapathi M., Palumbo M.J., Ansari S.A., He Q., Tsui K., Nislow C., Morse R.H. (2011). Extensive role of the general regulatory factors, Abf1 and Rap1, in determining genome-wide chromatin structure in budding yeast. Nucleic Acids Res..

[13] Miyake T., Loch C.M., Li R. (2002). Identification of a multifunctional domain in autonomously replicating sequence-binding factor 1 required for transcriptional activation, DNA replication, and gene silencing. Mol. Cell Biol..

[14] McGuffee S.R., Smith D.J., Whitehouse I. (2013). Quantitative, genome-wide analysis of eukaryotic replication initiation and termination. Mol. Cell.

[15] Poloumienko A., Dershowitz A., De J., Newlon C.S. (2001). Completion of replication map of Saccharomyces cerevisiae chromosome III. Mol. Biol. Cell.

[16] Berbenetz N.M., Nislow C., Brown G.W. (2010). Diversity of eukaryotic DNA replication origins revealed by genome-wide analysis of chromatin structure. PLoS Genet..

[17] Eaton M.L., Galani K., Kang S., Bell S.P., MacAlpine D.M. (2010). Conserved nucleosome positioning defines replication origins. Genes Dev..

[18] Thoma F., Bergman L.W., Simpson R.T. (1984). Nuclease digestion of circular TRP1ARS1 chromatin reveals positioned nucleosomes separated by nuclease-sensitive regions. J. Mol. Biol..

[19] Lipford J.R., Bell S.P. (2001). Nucleosomes positioned by ORC facilitate the initiation of DNA replication. Mol. Cell.

[20] Unnikrishnan A., Gafken P.R., Tsukiyama T. (2010). Dynamic changes in histone acetylation regulate origins of DNA replication. Nat. Struct. Mol. Biol..

[21] Aparicio J.G., Viggiani C.J., Gibson D.G., Aparicio O.M. (2004). The Rpd3-Sin3 histone deacetylase regulates RT and enables intra-S origin control in Saccharomyces cerevisiae. Mol. Cell Biol..

[22] Vogelauer M., Rubbi L., Lucas I., Brewer B.J., Grunstein M. (2002). Histone acetylation regulates the time of replication origin firing. Mol. Cell.

[23] McCarroll R.M., Fangman W.L. (1988). Time of replication of yeast centromeres and telomeres. Cell.

[24] Hafner L., Lezaja A., Zhang X., Lemmens L., Shyian M., Albert B., Follonier C., Nunes J.M., Lopes M., Shore D., Mattarocci S. (2018). Rif1 binding and control of chromosome-internal DNA replication origins is limited by telomere sequestration. Cell Rep..

[25] Peace J.M., Ter-Zakarian A., Aparicio O.M. (2014). Rif1 regulates initiation timing of late replication origins throughout the S. cerevisiae genome. PLoS One.

[26] Ferguson B.M., Fangman W.L. (1992). A position effect on the time of replication origin activation in yeast. Cell.

[27] Friedman K.L., Diller J.D., Ferguson B.M., Nyland S.V., Brewer B.J., Fangman W.L. (1996). Multiple determinants controlling activation of yeast replication origins late in S phase. Genes Dev..

[28] Aparicio O.M. (2013). Location, location, location: it’s all in the timing for replication origins. Genes Dev..

[29] Yamazaki S., Hayano M., Masai H. (2013). Replication timing regulation of eukaryotic replicons: Rif1 as a global regulator of replication timing. Trends Genet..

[30] Heun P., Laroche T., Shimada K., Furrer P., Gasser S.M. (2001). Chromosome dynamics in the yeast interphase nucleus. Science.

[31] Zhang H., Petrie M.V., He Y., Peace J.M., Chiolo I.E., Aparicio O.M. (2019). Dynamic relocalization of replication origins by Fkh1 requires execution of DDK function and Cdc45 loading at origins. Elife.

[32] Gauchier M., van Mierlo G., Vermeulen M., Déjardin J. (2020). Purification and enrichment of specific chromatin loci. Nat. Methods.

[33] Griesenbeck J., Boeger H., Strattan J.S., Kornberg R.D. (2003). Affinity purification of specific chromatin segments from chromosomal loci in yeast. Mol. Cell Biol..

[34] Hamperl S., Brown C.R., Garea A.V., Perez-Fernandez J., Bruckmann A., Huber K., Wittner M., Babl V., Stoeckl U., Deutzmann R. (2014). Compositional and structural analysis of selected chromosomal domains from Saccharomyces cerevisiae. Nucleic Acids Res..

[35] Raghuraman M.K., Winzeler E.A., Collingwood D., Hunt S., Wodicka L., Conway A., Lockhart D.J., Davis R.W., Brewer B.J., Fangman W.L. (2001). Replication dynamics of the yeast genome. Science.

[36] Batrakou D.G., Heron E.D., Nieduszynski C.A. (2018). Rapid high-resolution measurement of DNA replication timing by droplet digital PCR. Nucleic Acids Res..

[37] Fernández-Cid A., Riera A., Tognetti S., Herrera M.C., Samel S., Evrin C., Winkler C., Gardenal E., Uhle S., Speck C. (2013). An ORC/Cdc6/MCM2-7 complex is formed in a multistep reaction to serve as a platform for MCM double-hexamer assembly. Mol. Cell.

[38] Rossi M.J., Kuntala P.K., Lai W.K.M., Yamada N., Badjatia N., Mittal C., Kuzu G., Bocklund K., Farrell N.P., Blanda T.R. (2021). A high-resolution protein architecture of the budding yeast genome. Nature.

[39] Das S.P., Borrman T., Liu V.W.T., Yang S.C.-H., Bechhoefer J., Rhind N. (2015). Replication timing is regulated by the number of MCMs loaded at origins. Genome Res..

[40] Dukaj L., Rhind N. (2021). The capacity of origins to load MCM establishes replication timing patterns. PLoS Genet..

[41] Donovan S., Harwood J., Drury L.S., Diffley J.F. (1997). Cdc6p-dependent loading of Mcm proteins onto pre-replicative chromatin in budding yeast. Proc. Natl. Acad. Sci. USA.

[42] Bell S.P., Stillman B. (1992). ATP-dependent recognition of eukaryotic origins of DNA replication by a multiprotein complex. Nature.

[43] Huo L., Wu R., Yu Z., Zhai Y., Yang X., Chan T.C., Yeung J.T.F., Kan J., Liang C. (2012). The Rix1 (Ipi1p-2p-3p) complex is a critical determinant of DNA replication licensing independent of their roles in ribosome biogenesis. Cell Cycle.

[44] Jenni S., Harrison S.C. (2018). Structure of the DASH/Dam1 complex shows its role at the yeast kinetochore-microtubule interface. Science.

[45] Miranda J.J.L., King D.S., Harrison S.C. (2007). Protein arms in the kinetochore-microtubule interface of the yeast DASH complex. Mol. Biol. Cell.

[46] Westermann S., Avila-Sakar A., Wang H.-W., Niederstrasser H., Wong J., Drubin D.G., Nogales E., Barnes G. (2005). Formation of a dynamic kinetochore- microtubule interface through assembly of the Dam1 ring complex. Mol. Cell.

[47] Costanzo M., VanderSluis B., Koch E.N., Baryshnikova A., Pons C., Tan G., Wang W., Usaj M., Hanchard J., Lee S.D. (2016). A global genetic interaction network maps a wiring diagram of cellular function. Science.

[48] Eriksson P.R., Clark D.J. (2021). The yeast ISW1b ATP-dependent chromatin remodeler is critical for nucleosome spacing and dinucleosome resolution. Sci. Rep..

[49] Fang D., Lengronne A., Shi D., Forey R., Skrzypczak M., Ginalski K., Yan C., Wang X., Cao Q., Pasero P. (2017). Dbf4 recruitment by forkhead transcription factors defines an upstream rate-limiting step in determining origin firing timing. Genes Dev..

[50] Müller C.A., Boemo M.A., Spingardi P., Kessler B.M., Kriaucionis S., Simpson J.T., Nieduszynski C.A. (2019). Capturing the dynamics of genome replication on individual ultra-long nanopore sequence reads. Nat. Methods.

[51] Poli J., Gerhold C.-B., Tosi A., Hustedt N., Seeber A., Sack R., Herzog F., Pasero P., Shimada K., Hopfner K.-P. (2016). Mec1, INO80, and the PAF1 complex cooperate to limit transcription replication conflicts through RNAPII removal during replication stress. Genes Dev..

[52] Morawska M., Ulrich H.D. (2013). An expanded tool kit for the auxin-inducible degron system in budding yeast. Yeast.

[53] Duan Z., Andronescu M., Schutz K., McIlwain S., Kim Y.J., Lee C., Shendure J., Fields S., Blau C.A., Noble W.S. (2010). A three-dimensional model of the yeast genome. Nature.

[54] Knott S.R.V., Peace J.M., Ostrow A.Z., Gan Y., Rex A.E., Viggiani C.J., Tavaré S., Aparicio O.M. (2012). Forkhead transcription factors establish origin timing and long-range clustering in S. cerevisiae. Cell.

[55] Lõoke M., Kristjuhan K., Värv S., Kristjuhan A. (2013). Chromatin-dependent and -independent regulation of DNA replication origin activation in budding yeast. EMBO Rep..

[56] Ostrow A.Z., Nellimoottil T., Knott S.R.V., Fox C.A., Tavaré S., Aparicio O.M. (2014). Fkh1 and Fkh2 bind multiple chromosomal elements in the S. cerevisiae genome with distinct specificities and cell cycle dynamics. PLoS One.

[57] Vermeulen M., Déjardin J. (2020). Locus-specific chromatin isolation. Nat. Rev. Mol. Cell Biol..

[58] Reinapae A., Jalakas K., Avvakumov N., Lõoke M., Kristjuhan K., Kristjuhan A. (2017). Recruitment of Fkh1 to replication origins requires precisely positioned Fkh1/2 binding sites and concurrent assembly of the pre-replicative complex. PLoS Genet..

[59] Martin R.M., Cardoso M.C. (2010). Chromatin condensation modulates access and binding of nuclear proteins. FASEB J.

[60] Hsieh T.H.S., Weiner A., Lajoie B., Dekker J., Friedman N., Rando O.J. (2015). Mapping nucleosome resolution chromosome folding in yeast by micro-C. Cell.

[61] Grosche A., Hauser A., Lepper M.F., Mayo R., von Toerne C., Merl-Pham J., Hauck S.M. (2016). The proteome of native adult Müller Glial cells from Murine Retina. Mol. Cell. Proteomics.

[62] Wiśniewski J.R., Zougman A., Nagaraj N., Mann M. (2009). Universal sample preparation method for proteome analysis. Nat. Methods.

[63] Navarro P., Trevisan-Herraz M., Bonzon-Kulichenko E., Núñez E., Martínez-Acedo P., Pérez-Hernández D., Jorge I., Mesa R., Calvo E., Carrascal M. (2014). General statistical framework for quantitative proteomics by stable isotope labeling. J. Proteome Res..

