# Supplementary material for: Single-copy locus proteomics of early- and late-firing DNA replication origins identifies a role of Ask1/DASH complex in replication timing control
Source: Cell Rep. 2023 Jan 25;42(2):112045. doi: 10.1016/j.celrep.2023.112045 (PMC9989823; doi:10.1016/j.celrep.2023.112045)
Supplement: Document S1. Figures S1–S10 [file mmc1.pdf]

Cell Reports, Volume *42*

Supplemental information

Single-copy locus proteomics of early- and late-firing DNA replication origins identifies a role of Ask1/DASH complex in replication timing control

Matthias Weiβ, Anna Chanou, Tamas Schauer, Andrey Tvardovskiy, Stefan Meiser, Ann-Christine König, Tobias Schmidt, Elisabeth Kruse, Henning Ummethum, Manuel Trauner, Marcel Werner, Maxime Lalonde, Stefanie M. Hauck, Antonio Scialdone, and Stephan Hamperl

SUPPLEMENTARY INFORMATION

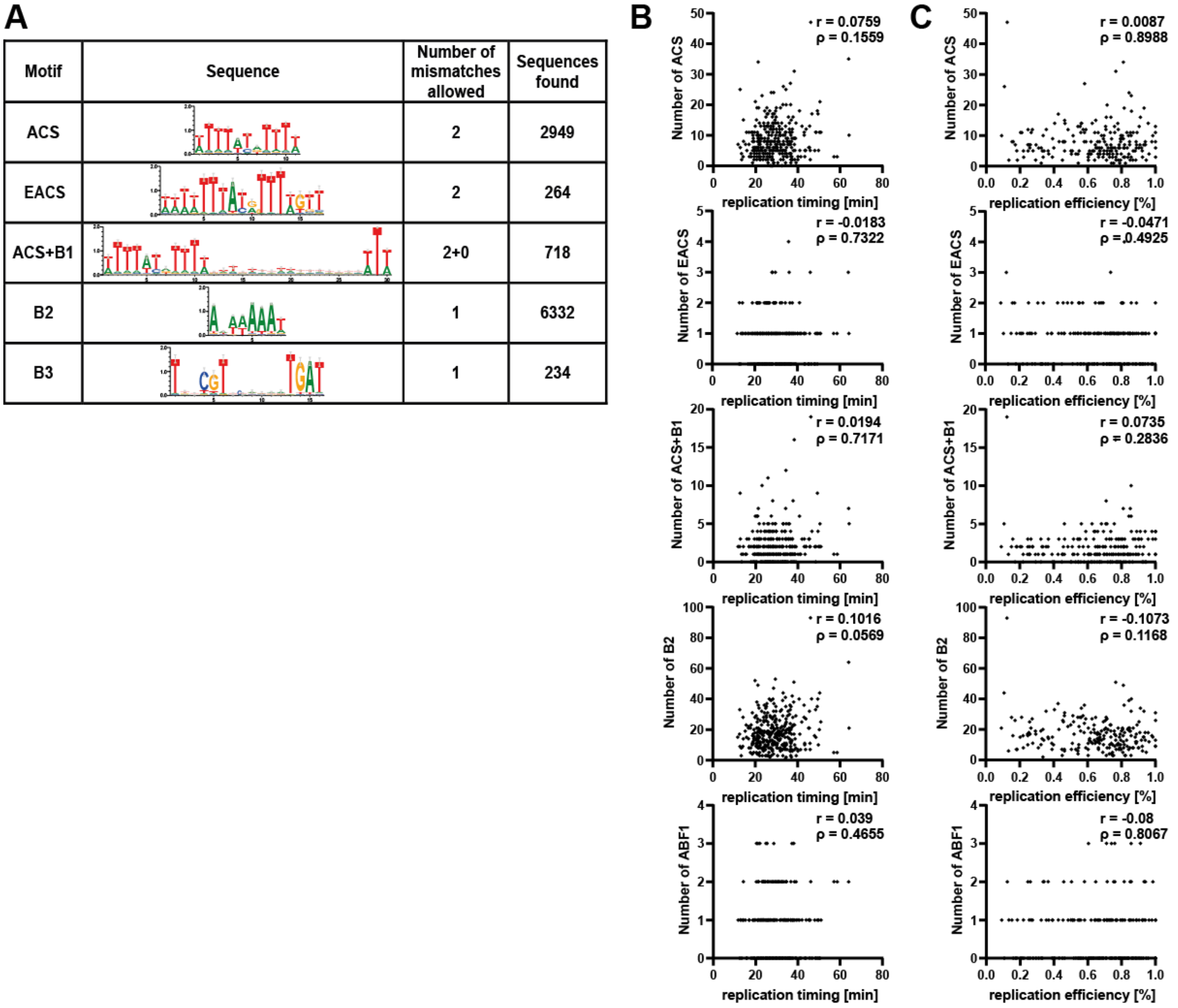

Figure S1, Related to Figure 1. DNA sequence features of yeast replication origins do not correlate with their replication timing and efficiency

A) 352 annotated yeast origins (SGD) were searched for the presence of specific DNA elements (ACS = WTTTAYRTTTW, eACS = WWWWTTTAYRTTTWGTT, ACS+B1 = WTTTAYRTTTWnnnnnnnnnnnnnnnWTW, B2 = ANWWAAAT, B3 = TnnCGTnnnnnnTGAT). The table indicates the number of the respective consensus sequences found based on the number of allowed mismatches. The DNA sequence logo shows the base distribution of the consensus sequences found at the replication origins. **B)** The frequency of the

respective DNA motifs at each origin was correlated with the known replication timing of each individual origin. r = Spearman’s Correlation Coefficient, p = p-value. **C)** Similarly, the frequency of the respective DNA motifs was correlated to available replication efficiency data. r = Spearman’s Correlation Coefficient, p = p-value.

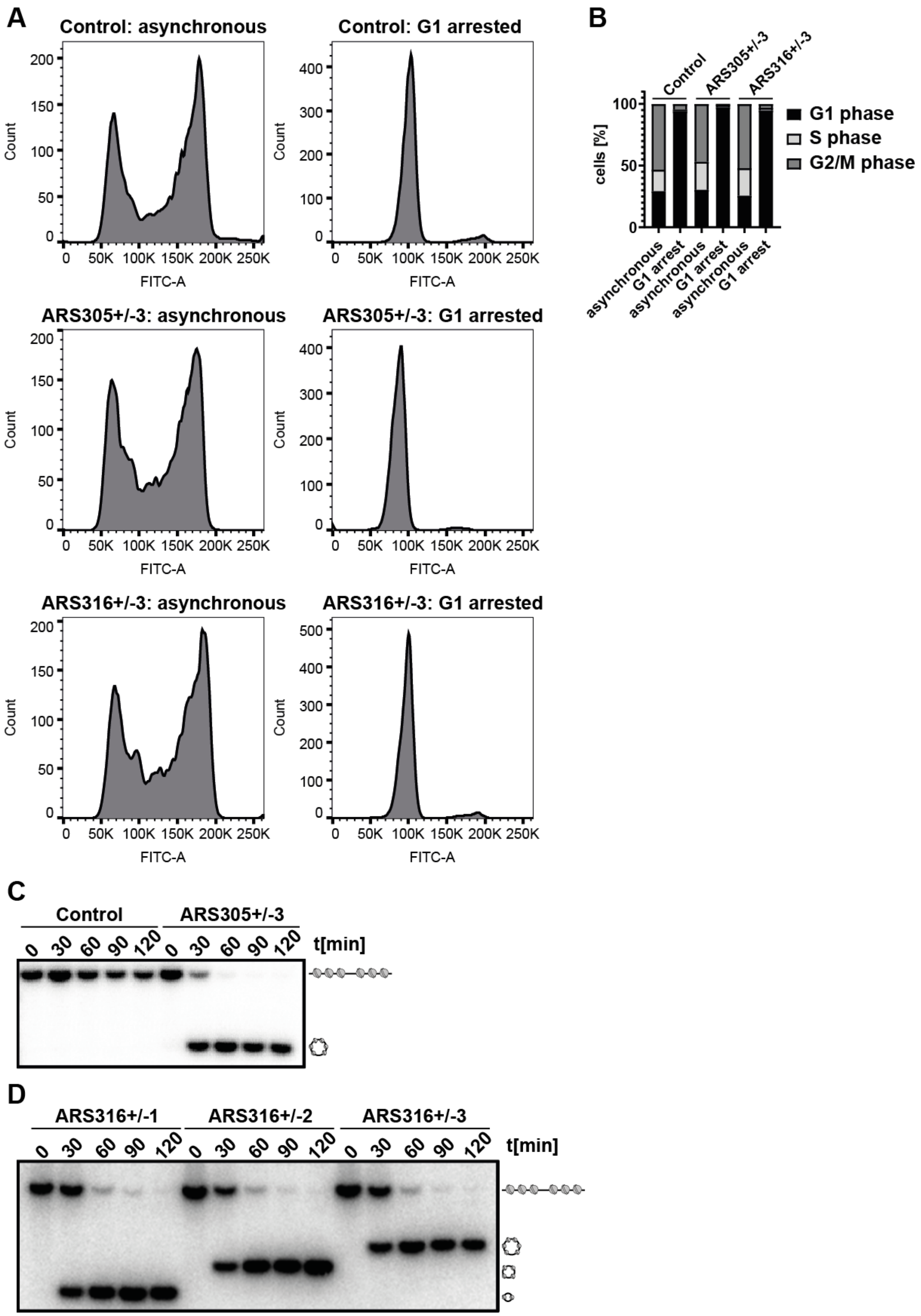
A
Control: asynchronous
Control: G1 arrested
ARS305+/-3: asynchronous
ARS305+/-3: G1 arrested
ARS316+/-3: asynchronous
ARS316+/-3: G1 arrested
Count
FITC-A
0
50K
100K
150K
200K
250K
B
Control
ARS305+/-3
ARS316+/-3
cells [%]
asynchronous
G1 arrest
G1 phase
S phase
G2/M phase
C
Control
ARS305+/-3
t[min]
0
30
60
90
120
D
ARS316+/-1
ARS316+/-2
ARS316+/-3
t[min]

Figure S2, Related to Figure 1. Generation and characterization of yeast strains competent for site-specific recombination and LexA affinity purification

A) Yeast strains competent for site-specific recombination Y0065 (ARS305+/-3) and Y0069 (ARS316+/-3) and Control strain Y0066 without RS-LEXA sites were grown to logarithmic phase. Subsequently, alpha factor (50ng/ml) was added to the cultures. Samples for FACS analysis were taken from asynchronous 2h alpha factor treated cultures. **B)** Distribution of G1, S, and G2 phases in each of the profiles from A) (n = 1). **C)** Yeast strain Y0065 (ARS305+/-3) was grown in YPR medium to logarithmic phase and then arrested in G1 phase in the presence of 2% Galactose to induce recombination. Genomic DNA samples were taken at the indicated timepoints, linearized with ClaI to allow visualization of the recombined and unrecombined genomic ARS305 locus by Southern blot analysis (n = 1). **(D)** Yeast strains Y0038 (ARS316+/-1), Y0039 (ARS316+/-2), and Y0040 (ARS316+/-3) were grown in YPR medium to logarithmic phase and then 2% Galactose was added to induce recombination. Genomic DNA samples were taken at the indicated timepoints, linearized with BstBI to allow visualization of the recombined and unrecombined genomic ARS316 locus by Southern blot analysis (n = 1).

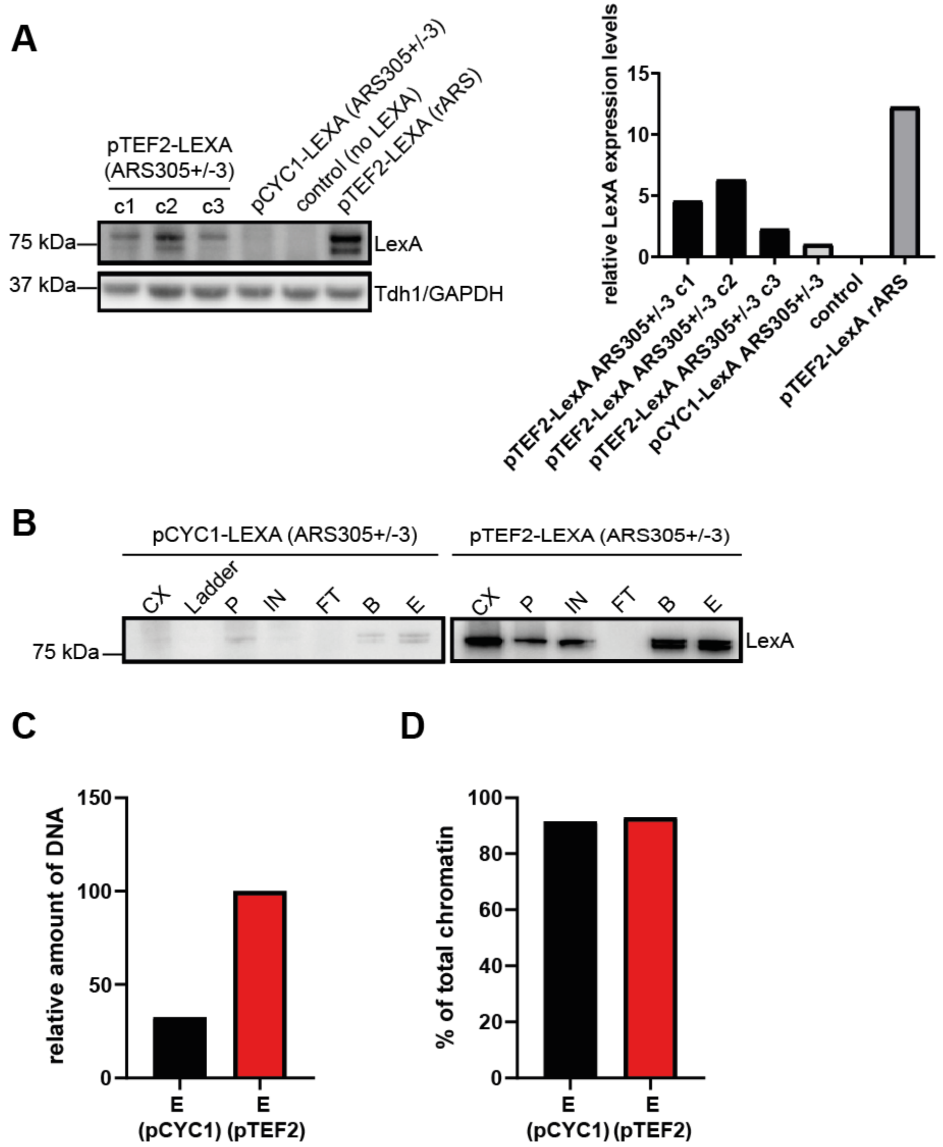

Figure S3, Related to Figure 1. TEF2 promoter-mediated expression levels of LexA-TAP are required for efficient pulldown of native chromatin domains

A) Levels of LexA-TAP protein was examined for indicated yeast strains utilizing different constitutive promoters for LexA expression. Three independent clones of ARS305+/-3 strains (Y0065 c1, c2 and c3) as well as a strain where the ribosomal ARS (rARS) is flanked by RS sites (Y0008) utilize a TEF2 promoter for expression of LexA-TAP. Another ARS305+/-3 strain expressing LexA under control of the weak CYC1 promoter (Y0037) was compared to a control strain without LexA expression (Y0034, no LEXA). Protein samples of total cell extracts were subjected to Western blot analysis using antibodies against LexA or Tdh1/GAPDH as loading

control. Bar graph on the right depicts quantification of the Western blot results (n = 1). **B)** LexA affinity purification was performed for yeast strain Y0037 (ARS305+/-3) which expresses LexA under control of the CYC1 promoter as well as Y0065 (ARS305+/-3) which expresses LexA under control of the TEF2 promoter. Protein samples were taken for each of the fractions of the purification process as shown in **Figure 1C** (0.1% for CX, P, IN, FT and 1% for B and E) and subsequently analyzed by Western blot analysis. **C)** DNA samples from the eluates of the affinity purifications in B) were taken (2.5%). DNA was extracted and analyzed by qPCR in order to monitor the amount of ARS305 present in the eluates (n = 1). **D)** Using the eluate samples from C), the fold enrichment of ARS305 compared to the PDC1 locus was calculated. Using the fold-enrichment values and factoring in the size of the total yeast genome (~12,000kb), the proportion of total DNA present in the final eluates derived from the targeted replication origin domain (~ 1kb) was calculated.

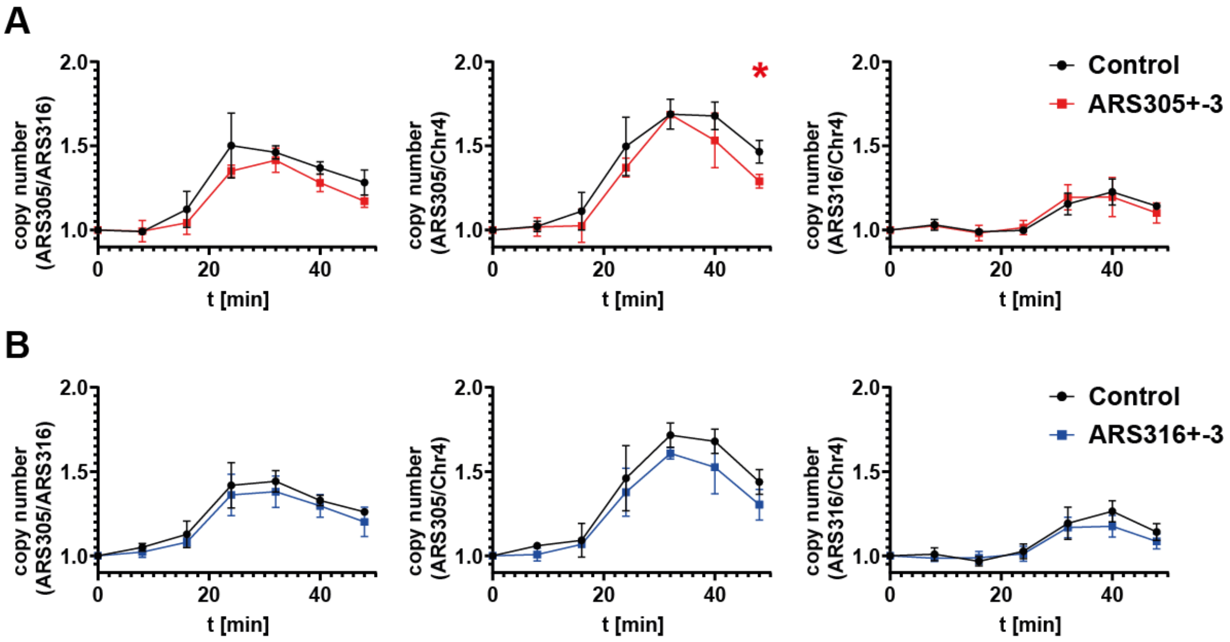

Figure S4, Related to Figure 1. Integration of RS- and LexA-binding sites does not affect the replication profile of the modified origins.

(A-B) Analysis of the replication timing of ARS305 and ARS316 for the strains where RS-sites and LexA binding sites were integrated next to ARS305+/-3 (Y0065) (A) or ARS316+/-3 (Y0069) (B) in comparison to a parental control strain (Y0066) that does not have RS- and LEXA- binding sites integrated in its genome. Samples for genomic DNA extraction were taken at the indicated timepoints for copy number analysis by qPCR to determine the relative replication timing of depicted loci. The plots show the average copy number ratios of early (ARS305) to late replicating regions (ARS316, Chr 4) with standard deviation from n = 3 biological replicates (* indicates statistical significance $p < 0.05$, unpaired t-test).

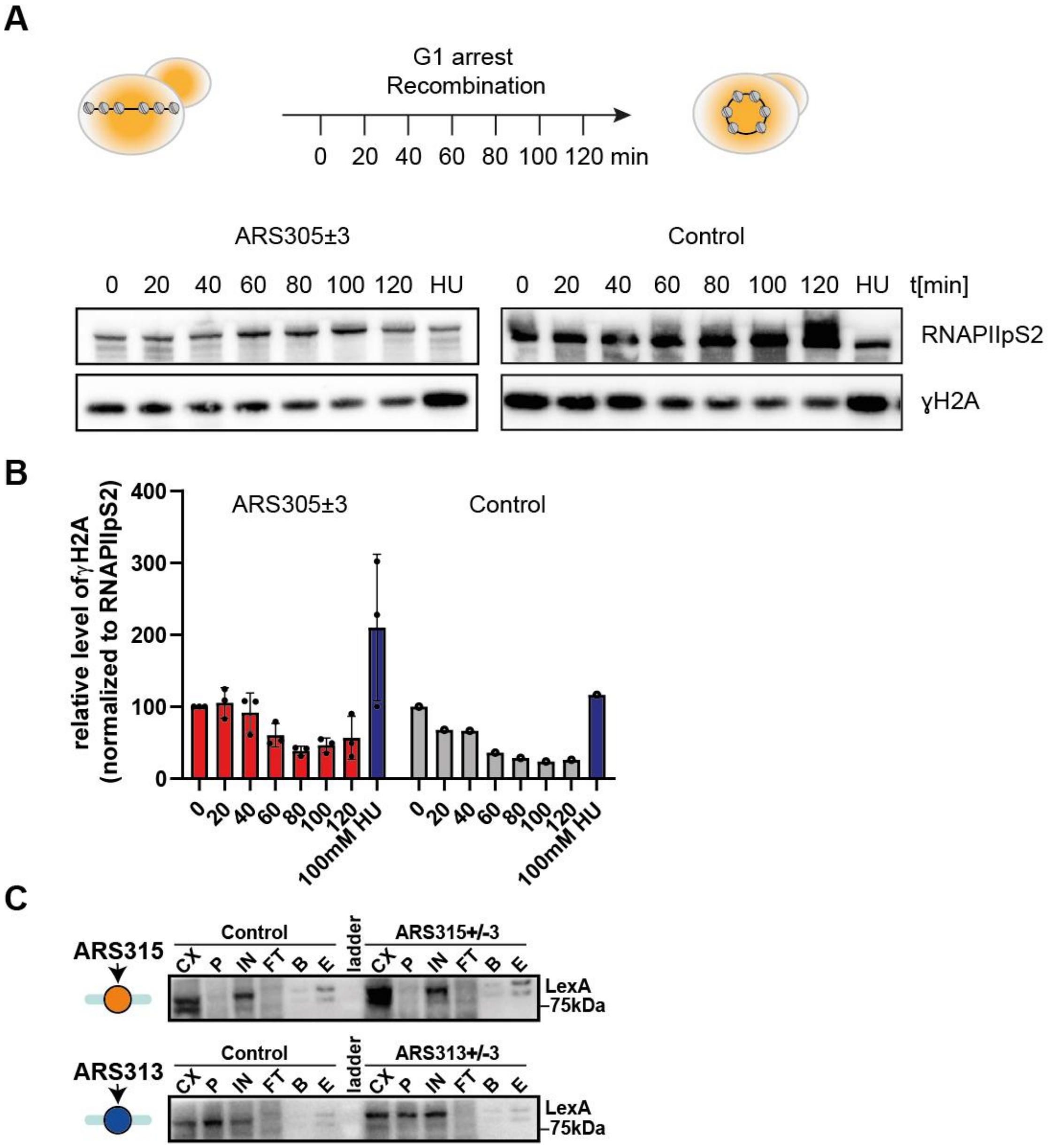

Figure S5, Related to Figure 1. Site-specific recombination does not induce detectable levels of DNA damage

A) Experimental outline for γH2A detection upon recombination induction. Yeast cells Y0037 (ARS305+/-3) and parental Control strain Y0034 without RS- and LEXA-binding sites were grown in YPR medium to logarithmic phase and then arrested with alpha factor in G1 phase in the presence of 2% Galactose to induce recombination. Protein samples were taken every 20min and analyzed by Western blot. As a positive control, cells were treated with 100mM hydroxyurea (HU) for 1h. Western blot analysis using antibodies against H2ApS129 (γH2A)

and RNAPII as a loading control. **B)** The bar plot shows the mean γH2A levels with standard deviation from n = 3 biological replicates for Y0037 and n = 1 biological replicate for Y0034. **C)** The LexA affinity purification was performed for yeast strains Y0091 (ARS315+/-3) and Y0094 (ARS313+/-3) as well as Y0066 (Control), a strain that also expresses LexA and R-Recombinase, but does not contain RS and LEXA sites integrated in the genome (Control). Protein samples were taken for each of the fractions of the purification process shown in Figure 1C) (0.1% for CX, P, IN, FT and 1% for B and E) and subjected to Western blot analysis to follow the levels of LexA-TAP protein during the purification.

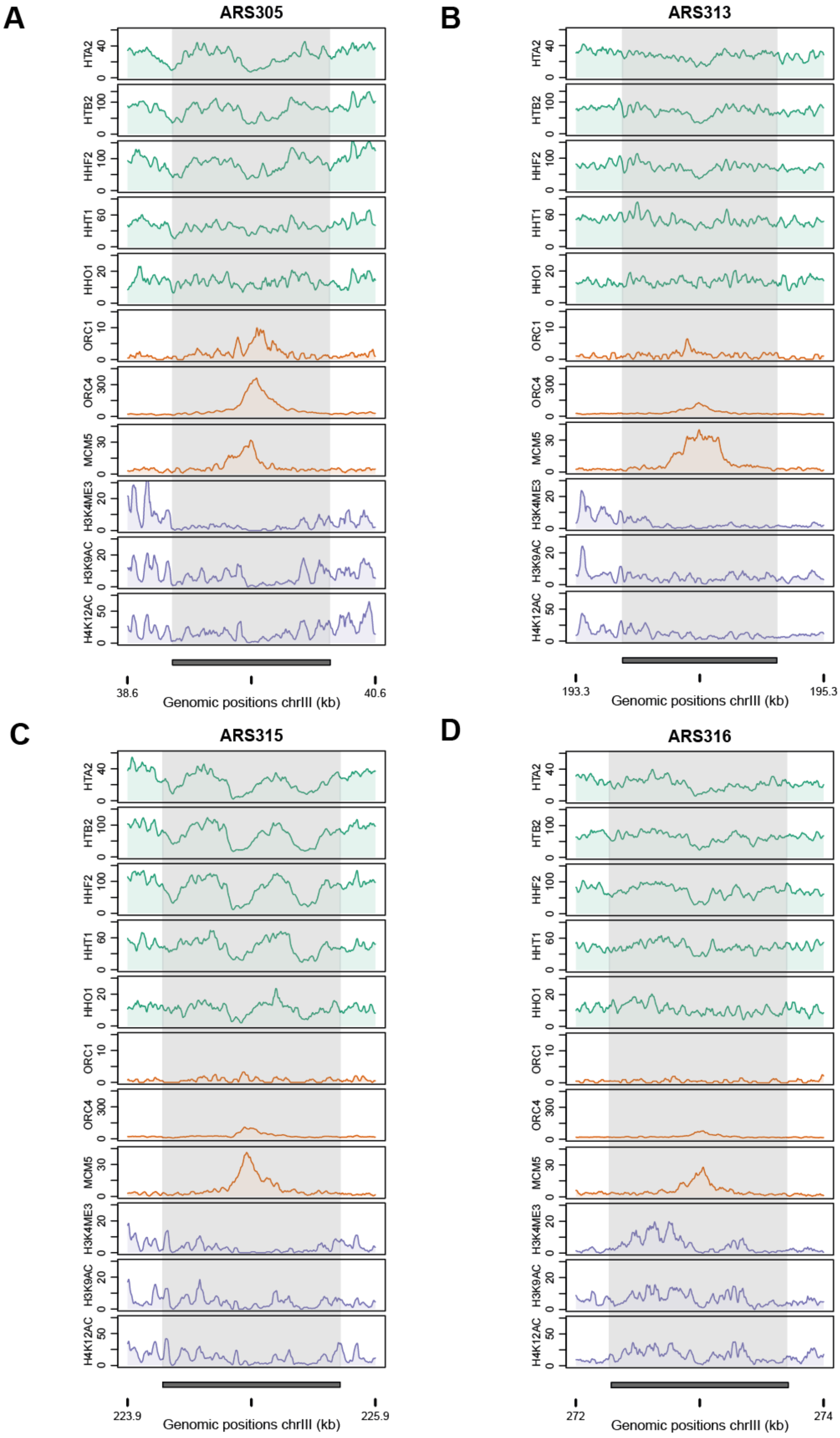

A
ARS305
HTA2
HTB2
HHF2
HHT1
HHO1
ORC1
ORC4
MCM5
H3K4ME3
H3K9AC
H4K12AC
38.6
40.6
Genomic positions chrIII (kb)
B
ARS313
193.3
195.3
Genomic positions chrIII (kb)
C
ARS315
223.9
225.9
Genomic positions chrIII (kb)
D
ARS316
272
274
Genomic positions chrIII (kb)

Figure S6, Related to Figure 2. ChIP-Exo chromatin profiles of the four selected origin regions.

A-D) ChIP-Exo profiles of canonical histones H2A (HTA2), H2B (HTB2), H4 (HHF2), H3 (HHT1), H1 (HHO1), replication factors ORC1, ORC4, MCM5 and histone PTMs H3K4me3, H3K9ac and H4K12ac) across the four selection origins ARS305, ARS313, ARS315 and ARS316. The grey box indicates the region of each origin that is flanked by RS sites and therefore part of the purified chromatin circles.

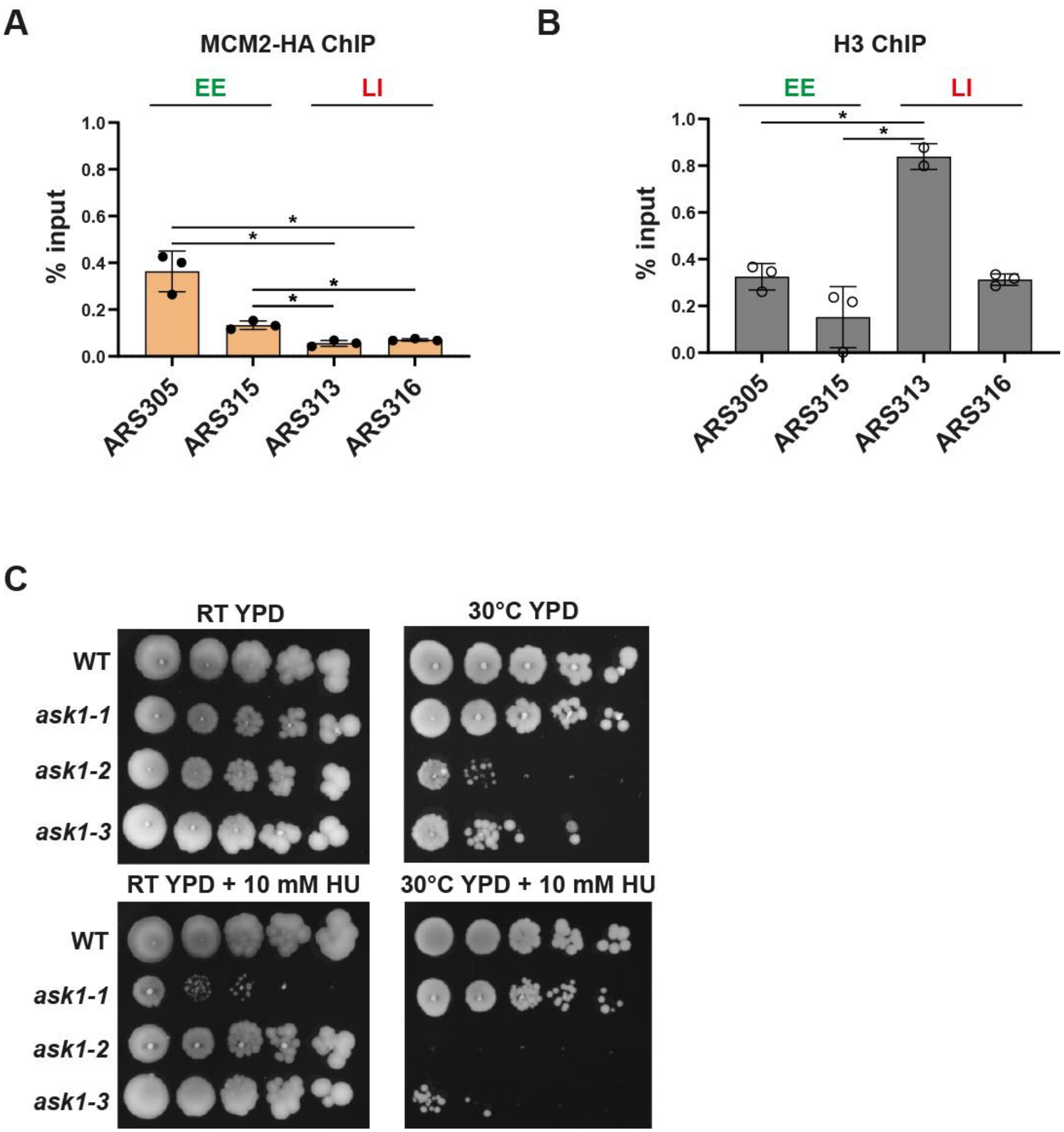

Figure S7, Related to Figures 2, 3. ChIP-qPCR analysis confirms the difference in MCM2-7 to histone ratios at EE and LI origins and Ask1 temperature-sensitive mutants are sensitive to HU-induced replication stress.

A) ChIP-qPCR analysis in yeast strain Y0124 expressing MCM2 as an HA-tagged allele at the indicated EE origins ARS305/ARS315 and LI origins ARS313/ARS316. An HA-antibody was

used to immunoprecipitate MCM2-HA at the indicated genomic regions. The bars indicate mean and standard deviations from 3 biological replicates (* indicates statistical significance $p < 0.05$, unpaired t-test). **B)** ChIP-qPCR analysis in yeast strain Y0001 at the indicated EE origins ARS305/ARS315 and LI origins ARS313/ARS316. A pan-H3 antibody was used to immunoprecipitate H3 at the indicated genomic regions. The bars indicate mean and standard deviations from 3 biological replicates (* indicates statistical significance $p < 0.05$, unpaired t-test). **C)** Spot tests of the three temperature-sensitive Ask1 mutants ask1-1 (Y0099), ask1-2 (Y0100), and ask1-3 (Y0101) together with the corresponding wildtype strain (Y0098). Growth inhibition was monitored on YPD plates of serial dilutions of the strains with or without 10mM HU and incubation for 3 days at the indicated temperatures (RT room temperature).

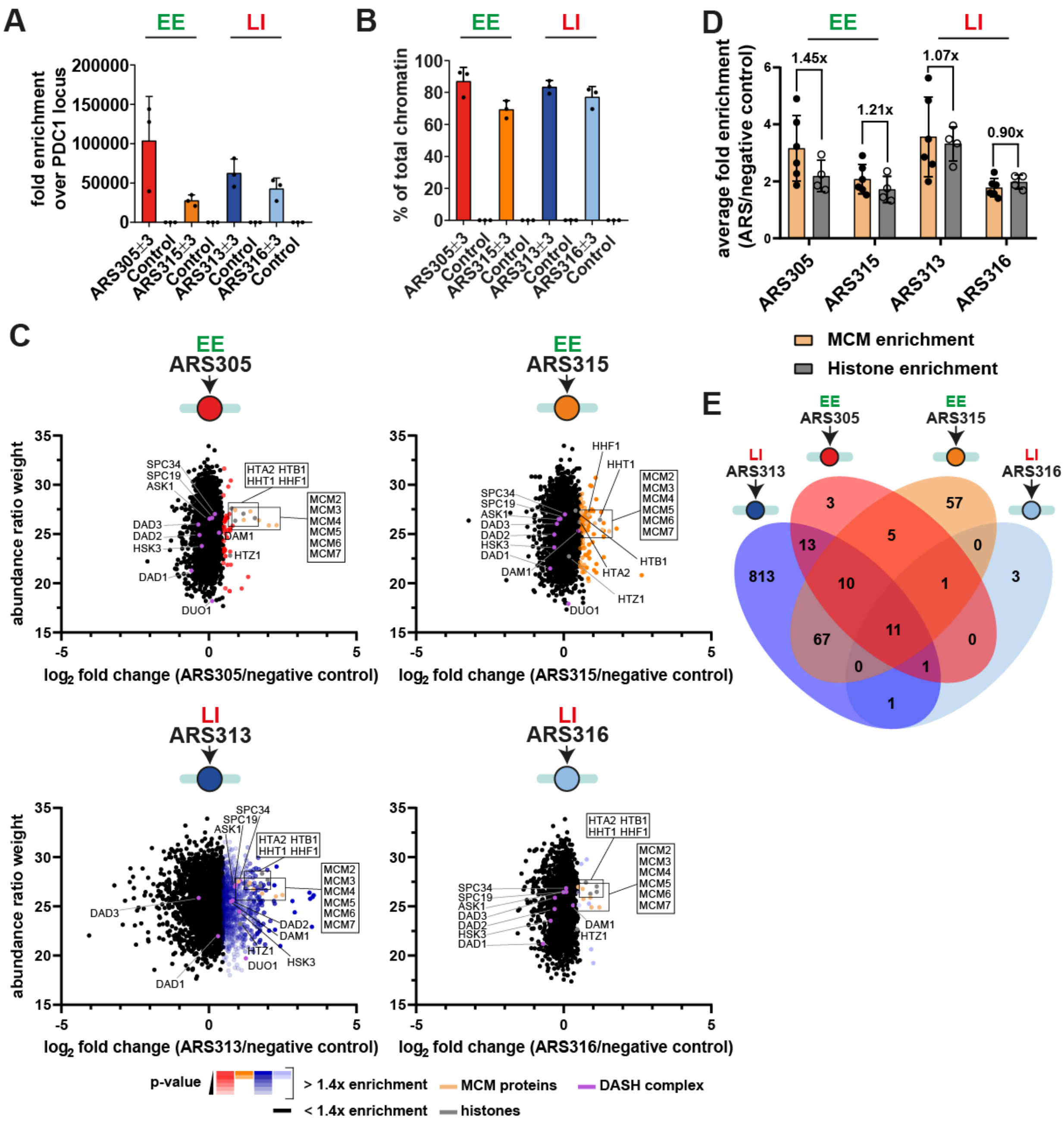

Figure S8, Related to Figure 2. LexA affinity purification of EE and LI origin chromatin domains under low-salt conditions of 150mM KCl

A) LexA affinity purifications were performed under low-salt conditions (150mM KCl) for all the strains proficient for site-specific recombination of the targeted replication origins (Y0065 (ARS305+/-3), Y0091(ARS315+/-3), Y0094 (ARS313+/-3), Y0069 (ARS316+/-3)) as well as one purification from the Control strain (Y0066). DNA samples from 3 biological replicates

were taken from the final eluate E (2.5%). DNA was extracted and analyzed by qPCR to monitor the fold-enrichment of the targeted replication origins compared to the PDC1 locus (n = 3 biological replicates). **B)** Using the fold-enrichment values from A) and factoring in the size of the total yeast genome (~12,000kb), the proportion of total DNA present in the final eluates derived from the targeted replication origin domain (~ 1kb) was calculated (n = 3 biological replicates). **C**) Scatter plots of abundance ratio weights vs. the average log2-fold enrichment of proteins at each of the respective replication origin purification (n = 3 biological replicates). Proteins of the MCM2-7 complex are shown in light orange, histones in grey and DASH complex subunits in purple. All proteins that were statistically enriched at least 1.4-fold over the control purification are colored according to the origin and decreasing p-value as indicated in the legend below. **D)** Bar plots representing the average enrichment of the four canonical histones (H2A, H2B, H3, H4) and the six MCM2-7 subunits (MCM2, MCM3, MCM4, MCM5, MCM6, MCM7) over the control purification for each respective replication origin. The bars indicate mean and standard deviations from each subunit of the complexes from 3 biological replicates. **E)** Venn diagram showing the overlap of proteins detected in the four replication origin purifications at low-salt conditions (150mM KCl). Only proteins at least 1.4-fold enriched over the negative control are included.

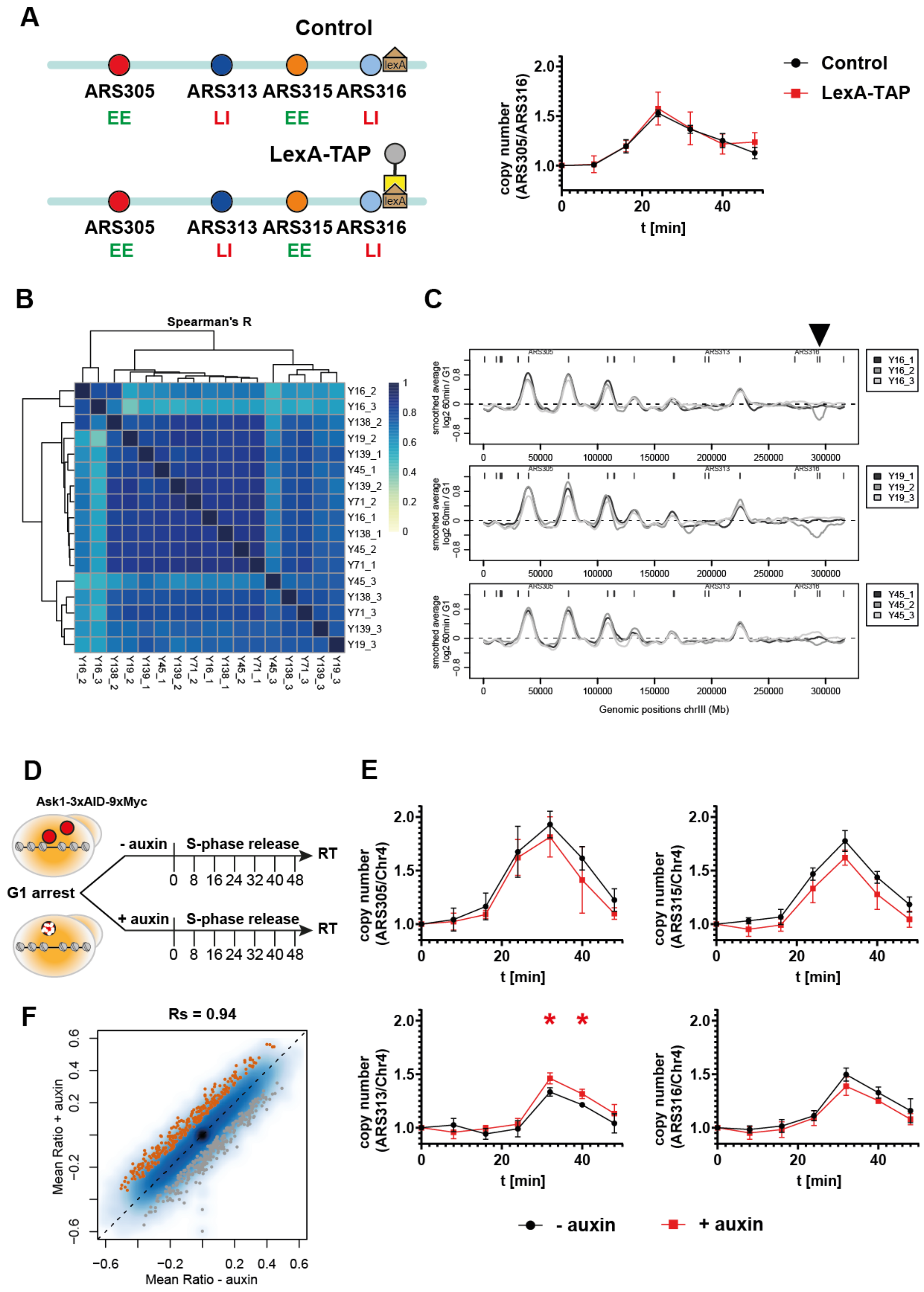
A
Control
ARS305
ARS313
ARS315
ARS316
EE
LI
EE
LI
lexA
LexA-TAP
copy number (ARS305/ARS316)
t [min]
Control
LexA-TAP
B
Spearman's R
Y16_2
Y16_3
Y138_2
Y19_2
Y139_1
Y45_1
Y139_2
Y71_2
Y16_1
Y138_1
Y45_2
Y71_1
Y45_3
Y138_3
Y71_3
Y139_3
Y19_3
C
smoothed average log2 60min / G1
Y16_1
Y16_2
Y16_3
Y19_1
Y19_2
Y19_3
Y45_1
Y45_2
Y45_3
Genomic positions chrIII (Mb)
D
Ask1-3xAID-9xMyc
G1 arrest
- auxin
+ auxin
S-phase release
RT
0 8 16 24 32 40 48
E
copy number (ARS305/Chr4)
copy number (ARS315/Chr4)
copy number (ARS313/Chr4)
copy number (ARS316/Chr4)
t [min]
- auxin
+ auxin
F
Rs = 0.94
Mean Ratio + auxin
Mean Ratio - auxin

Figure S9, Related to Figures 3, 4, 5. Targeted degradation of Ask1 protein in G1 advances replication timing of LI origin ARS313

A) Experimental outline for assessing the effect of tethering LexA-TAP at the LI replication origin ARS316. Yeast strains Y0019 (Control) and Y0069 (LexA-TAP) contain a cluster of 3x LEXA binding sites in close proximity to ARS316. Y0069 but not Y0019 expresses a LexA-TAP fusion protein that can bind to the 3xLEXA sites. Analysis of the replication timing by copy number analysis. Samples for genomic DNA extraction were taken at the indicated timepoints for copy number analysis by qPCR to determine the relative replication timing of depicted loci. The plots show the average copy number ratios of the origins ARS305 and ARS316 with standard deviation from n = 1 biological replicates. **B)** Spearman correlation matrix of the replication timing profiles of all biological replicates of the Control (Y16, Y19 and Y45) and tethering strains (Y71, Y138, Y139) at the 24min release timepoint and a genomic bin size of 1000bp. **C)** Replication profiles of chromosome III at 60min +HU after release into S-phase of the individual biological replicates of the control strains Y16, Y19 and Y45 and a bin size of 1000bp. The black arrow indicates a region with high variability among biological replicates. **D)** Schematic outline of the G1 arrest and release experiment. Y0123 was grown to logarithmic phase and arrested in G1 phase by alpha factor treatment. Cells were then cultured for 30min in the presence or absence of 1mM auxin before release into S phase by addition of 125 U Pronase. Samples were then taken at the indicated timepoints for replication timing (RT) analysis. **E)** Samples for genomic DNA extraction were taken at the indicated timepoints for copy number analysis by qPCR to determine the relative replication timing of depicted loci. The plots show the average copy number ratios of early (ARS305 and ARS315) and late origins (ARS313 and ARS316) in comparison to a late replicating region on Chr. 4 with standard deviation from n=3 biological replicates (*indicates statistical significance $p < 0.05$, unpaired t-test). **F)** Smoothed scatter plot showing correlation of RT values between minus auxin and plus auxin treated samples at 24min timepoint after S phase release and 500bp bin size. Orange and grey dots indicate genomic bins that were significantly increased or decreased in the two conditions. Rs is Spearman's R coefficient.

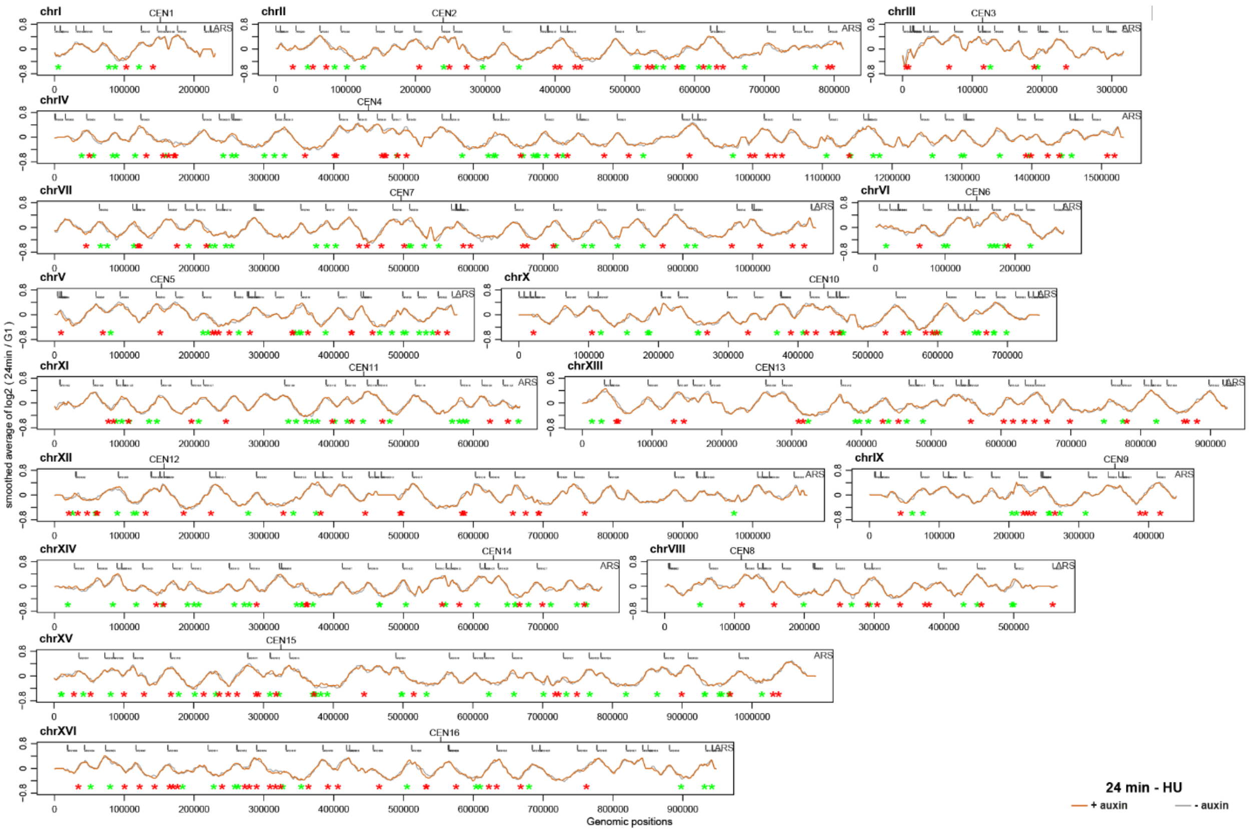

Figure S10, Related to Figure 5. Replication profiles of all yeast chromosomes 24min after S-phase release

A) Replication profiles of all chromosomes at 24min after release into S-phase after degrading Ask1 by addition of auxin in the strain Y0123. Regions that significantly increased or decreased replication timing were obtained using Welch two sample t-test (unequal variances) in each genomic 500 bp bin with a p-value cutoff of 0.05 and a mean difference of at least 0.1 and indicated with green or red asterisks, respectively.
